# Pioneering function of Isl1 in the epigenetic control of cardiomyocyte cell fate

**DOI:** 10.1038/s41422-019-0168-1

**Published:** 2019-04-25

**Authors:** Rui Gao, Xingqun Liang, Sirisha Cheedipudi, Julio Cordero, Xue Jiang, Qingquan Zhang, Luca Caputo, Stefan Günther, Carsten Kuenne, Yonggang Ren, Shoumo Bhattacharya, Xuejun Yuan, Guillermo Barreto, Yihan Chen, Thomas Braun, Sylvia M. Evans, Yunfu Sun, Gergana Dobreva

**Affiliations:** 1 Max-Planck-Institute for Heart and Lung Research, Bad Nauheim, Germany; 2 Department of Anatomy and Developmental Biology, CBTM, Medical Faculty Mannheim, Heidelberg University, Mannheim, Germany; 3 European Center for Angioscience (ECAS), Medical Faculty Mannheim, Heidelberg University, Mannheim, Germany; 4 Key Laboratory of Arrhythmia, Ministry of Education, East Hospital, Tongji University School of Medicine, Shanghai, 200120 China; 5 Department of Cardiovascular Medicine, University of Oxford, Oxford, UK; 6 Department of Medicine, Skaggs School of Pharmacy, University of California, San Diego, La Jolla, CA 92093 USA; 7 Medical Faculty, University of Frankfurt, 60590 Frankfurt am Main, Germany

**Keywords:** Epigenetic memory, Chromatin remodelling, Developmental biology

## Abstract

Generation of widely differing and specialized cell types from a single totipotent zygote involves large-scale transcriptional changes and chromatin reorganization. Pioneer transcription factors play key roles in programming the epigenome and facilitating recruitment of additional regulatory factors during successive cell lineage specification and differentiation steps. Here we show that Isl1 acts as a pioneer factor driving cardiomyocyte lineage commitment by shaping the chromatin landscape of cardiac progenitor cells. Using an Isl1 hypomorphic mouse line which shows congenital heart defects, genome-wide profiling of Isl1 binding together with RNA- and ATAC-sequencing of cardiac progenitor cells and their derivatives, we uncover a regulatory network downstream of Isl1 that orchestrates cardiogenesis. Mechanistically, we show that Isl1 binds to compacted chromatin and works in concert with the Brg1-Baf60c-based SWI/SNF complex to promote permissive cardiac lineage-specific alterations in the chromatin landscape not only of genes with critical functions in cardiac progenitor cells, but also of cardiomyocyte structural genes that are highly expressed when Isl1 itself is no longer present. Thus, the Isl1/Brg1-Baf60c complex plays a crucial role in orchestrating proper cardiogenesis and in establishing epigenetic memory of cardiomyocyte fate commitment.

## Introduction

The differentiation of stem/progenitor cells into distinct lineages involves a coordinated series of large-scale transcriptional changes and chromatin reorganization. Tissue specific transcription factors cooperate with epigenetic modifiers to program the epigenome and establish cellular identity, which is further maintained by epigenetic regulatory mechanisms. To initiate cell programming, a special type of transcription factors, named pioneer transcription factors, engage developmentally silenced genes embedded in “closed” chromatin covered by nucleosomes.^1–4^ Pioneer factor binding on its own is not sufficient for gene activation, but it imparts competence for transcription by chromatin opening. Chromatin opening facilitates subsequent recruitment of additional transcription factors and other regulatory proteins, which work in concert to induce a cell-type-specific gene expression program during the successive steps involved in lineage specification and differentiation.^1–4^ During cardiogenesis, multiple transcription factors cooperate and are integrated in regulatory networks, which strictly control a transcriptional program that ensures proper heart development.^5–8^

Isl1, a LIM-homeodomain transcription factor is transiently expressed in second heart field (SHF) progenitor cells before their differentiation and integration into the heart tube.^9^ These cells are added to the arterial and venous poles of the heart tube allowing its continuous growth and complex morphogenetic patterning.^8–10^ Defects in specification, deployment and differentiation of SHF cardiac progenitor cells (CPCs) are largely responsible for the high rate of congenital cardiac abnormalities in humans, underscoring the importance of a more integrated understanding of the mechanisms driving SHF-mediated cardiogenesis.^11^ The key role of Isl1 in SHF development is evident from genetic studies in mice, showing that Isl1-deficient mouse embryos lack all structures derived from the SHF including the right ventricle (RV), the outflow tract (OFT) and large portions of the atria, since Isl1 is required for proliferation, survival, migration of SHF CPCs and their differentiation into the different cardiac lineages.^9,12–14^ Importantly, recent studies identified association of *ISL1* variants and deletion with congenital heart disease.^15–17^ Despite the critical role of Isl1 in cardiac development and disease, detailed insights into its molecular mode of action are critically missing.

The Brg1-based SWI/SNF complex acts as a global transcriptional regulator by altering chromatin structure and DNA accessibility. Brg1, the catalytic component of the complex, utilizes energy from ATP hydrolysis to disrupt or reposition nucleosomes, thereby activating or repressing transcription, depending on the inclusion or exclusion of specific accessory factors.^18^ Mice haploinsufficient for *Brg1* exhibit cardiac morphogenetic defects, suggesting a key role of Brg1 in heart development.^19^ The functional versatility of the Brg1-based SWI/SNF complex is highly determined by the dynamic assembly of BAF subunits, some of which show a cell-type-specific expression pattern. Consistently, depletion of the cardiac-specific subunit of the Brg1 complex *Baf60c* leads to severe cardiac abnormalities.^20^ Moreover, Baf60c mediates the interaction between the core cardiac transcription factors Tbx5, Nkx2-5, Gata4 and the Brg1 complex, thereby regulating expression of their target genes.^20^ Importantly, Baf60c was shown to promote cardiomyocyte fate and differentiation of non-cardiogenic mesoderm in concert with Gata4 and Tbx5 by facilitating the binding of Gata4 to its cardiac-specific target genes and inducing hierarchical downstream regulatory networks.^21^

Here we show that Isl1 acts as a pioneer factor in cardiomyocyte fate commitment by shaping the epigenetic landscape of CPCs. On the one hand, Isl1 binds to and regulates the expression of transcription factors, epigenetic modifiers and signaling molecules with critical functions and high expression in CPCs. On the other hand, in CPCs Isl1 also binds to cardiomyocyte structural genes and genes involved in cardiomyocyte function, well before these genes become highly expressed in differentiating cardiomyocytes. Our data further demonstrate that Isl1 binds to closed chromatin and works in concert with the Brg1-Baf60c-based SWI/SNF complex to induce permissive cardiac lineage-specific alterations in the chromatin landscape of CPCs enabling the subsequent activation of genes defining cardiomyocyte identity in cardiomyocytes, when Isl1 itself is switched off.

## Results

### *Isl1* hypomorphic embryos show defects in cardiac morphogenesis, cardiomyocyte differentiation and maturation

**Fig. 1:**
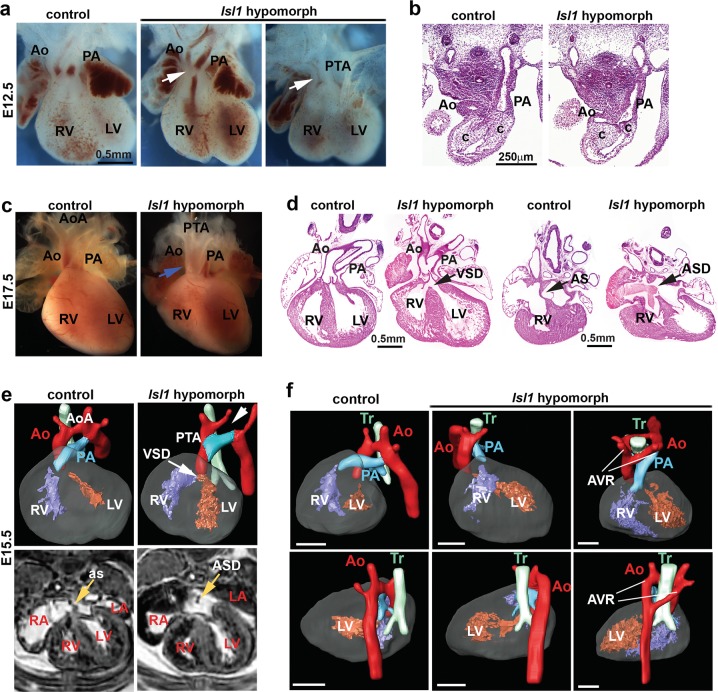
Reduced Isl1 expression leads to defects in cardiac morphogenesis. **a**, **b** Wholemount and histological analysis of E12.5 hearts of control and *Isl1* hypomorphs littermates showing impaired septation of the aorta (Ao) and pulmonary artery (PA) in *Isl1* hypomorphs. Examples of *Isl1* hypomorph showing partial septation of the aorta and the pulmonary artery with misaligned and hypoplastic aorta (**a**, middle panel, and **b**), and *Isl1* hypomorph showing persistent truncus arteriosus (PTA, arrow). Cushion, c; Aorta, Ao; left ventricle, LV; right ventricle, RV. **c** Macroscopic appearance of E17.5 control and *Isl1* hypomorphic heart with PTA (arrow). **d** Histological analysis of *Isl1* hypomorphic hearts at E17.5 showing atrial septal defect (ASD, arrow), ventricular septal defect (VSD, arrow) and PTA compared to control hearts. At E17.5, a proportion of the mutant hearts are dilated. Atrial septum, AS. **e**, **f** MRI and 3D reconstruction of control and *Isl1* hypomorphic hearts at E15.5 showing PTA, VSD and ASD (**e**) or other complex outflow tract phenotypes (**f**), e.g. right aortic arch (**f**, middle panel) and aortic vascular ring (AVR) (**f**, right panel). Aorta, Ao; aortic arch, AoA; pulmonary artery, PA; trachea, Tr; left ventricle, LV; right ventricle, RV; as, atrial septum; atrial septal defect (ASD), ventricular septal defect (VSD)

To investigate the mechanisms through which Isl1 regulates cardiogenesis, we utilized an *Isl1* knockout mouse line, which shows early embryonic lethality^9,22^ and an *Isl1* hypomorphic mouse line (Supplementary information, Fig. S1a–d),^23^ which survives until birth, allowing us to analyze the role of Isl1 in SHF structures that are dependent on Isl1, as well as during later stages of embryonic heart development. Consistent with human studies which identified *ISL1* variations and deletions contributing to congenital heart disease,^16,17^ all *Isl1* hypomorphic mice (*Isl1*-f;neo/f;neo) died shortly after birth with severe cardiac malformations. Wholemount and histological analyses of *Isl1* hypomorphic embryos at E12.5 (Fig. 1a, b) and E17.5 (Fig. 1c, d) revealed various degrees of cardiac outflow tract (OFT) septation abnormalities, including partial OFT septal defects with aortic stenosis and misalignment (Fig. 1a, middle panel, Fig. 1b) and persistent truncus arteriosus (PTA) (Fig. 1a, right panel, Fig. 1c, d). Nearly all *Isl1* hypomorphic mice presented ventricular septal defects (VSDs) and atrial septal defects (ASDs) (Fig. 1d). MRI and 3D reconstructions confirmed the presence of various cardiac outflow tract (OFT) abnormalities, VSDs and ASDs (Fig. 1e, f).

**Fig. 2:**
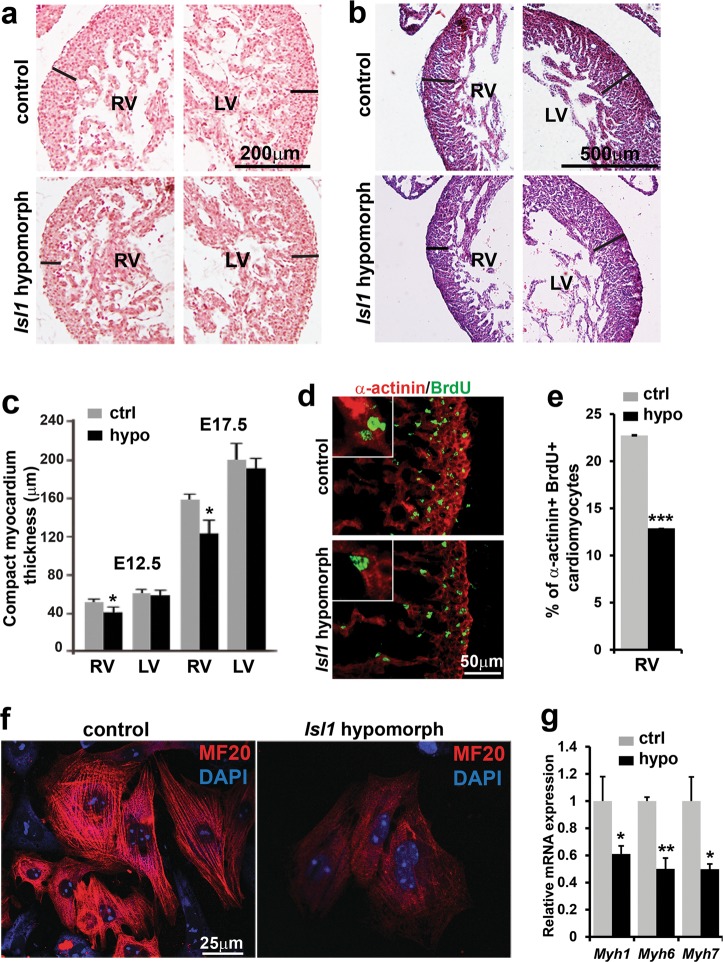
Reduced Isl1 expression leads to defects in cardiomyocyte proliferation, differentiation and maturation. **a**–**c** Reduced wall thickness of right ventricle in *Isl1* hypomorphic hearts compared to the control littermates at E12.5 (**a**, **c**) and E17.5 (**b**, **c**). Left ventricle wall thickness in *Isl1* hypomorphic hearts is largely comparable to the controls when mutant hearts are not significantly dilated. Data represent mean ± SEM, *n* = 6. **d** Co-immunostaining of control and *Isl1* hypomorphic right ventricles with BrdU and α-actinin at E12.5. **e** Percentage of BrdU-labeled cardiomyocytes (BrdU^+^/α-actinin^+^ cells) in RV free wall at E12.5, showing a marked reduction in the number of proliferating cardiomyocytes in *Isl1* hypomorphs. Data represent mean ± SEM, *n* = 4. **f** Markedly reduced MF20 staining and less prominent sarcomere structure in cardiomyocytes isolated from control and *Isl1* hypomorphic E15.5 hearts. **g** Relative *Myh1*, *Myh6* and *Myh7* mRNA expression in isolated *Isl1* hypomorphic cardiomyocytes compared to control cardiomyocytes. Statistical significance in this and all other Figs., unless otherwise stated, was determined by two tailed student *t*-test *p ≤ 0.05, **p ≤ 0.01, ***p ≤ 0.005

Detailed histological analysis revealed that the right ventricular compact myocardium of *Isl1* hypomorphic mice was thinner than that of control littermates at E12.5 (Fig. 2a, c) and E17.5 (Fig. 2b, c). In contrast, left ventricular wall thickness in *Isl1* hypomorphic mice was relatively normal in embryos with no significant ventricular dilation (Fig. 2a–c). Co-immunostaining for alpha-actinin and BrdU together with staining for MF20 and BrdU at E12.5 and E17.5 revealed significantly decreased cardiomyocyte proliferation in *Isl1* hypomorphic hearts and markedly decreased MF20 immunoreactivity (Fig. 2d, e; Supplementary information, Fig. S1e, f). Further, qPCR analysis for *Myh6* and *Myh7* as well as immunostaining for MF20 on E15.5 cardiomyocytes in culture revealed decreased expression of cardiac myosins and less prominent sarcomere structures (Fig. 2f, g), suggesting defects in differentiation of cardiac progenitors and in sarcomeric maturation.

### Isl1 orchestrates a complex gene regulatory network driving cardiogenesis

**Fig. 3:**
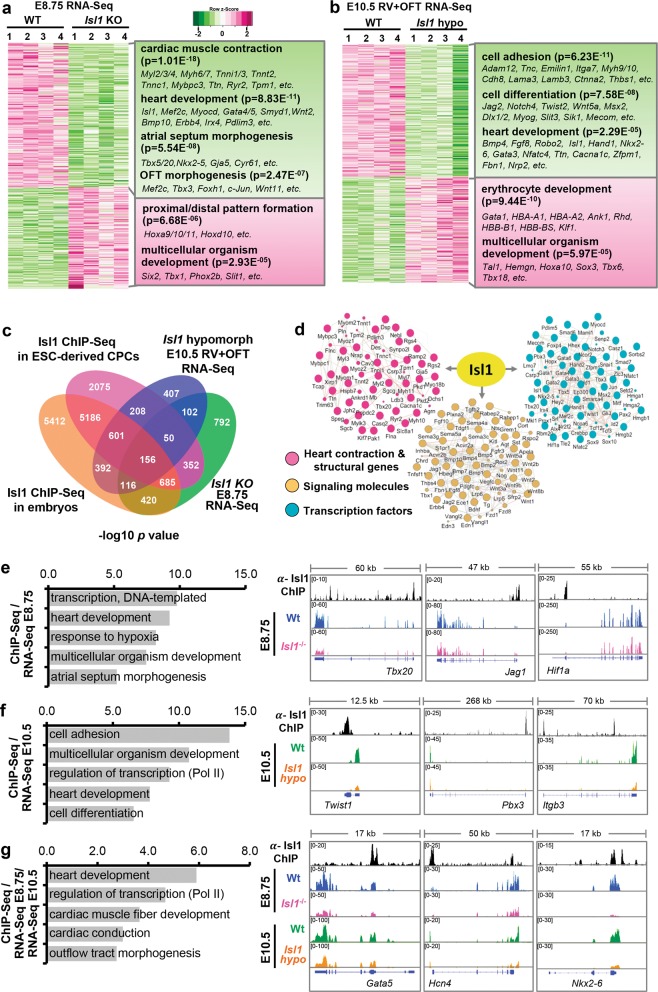
Isl1 orchestrates a complex gene regulatory network driving cardiogenesis. **a**, **b** Heatmap representation of RNA-Seq analysis of dissected pharyngeal mesoderm and hearts of wild-type and E8.75 *Isl1* knockout embryos (**a**) as well as dissected OFT and RV of E10.5 wild-type and *Isl1* hypomorphic embryos (*n* = 4, fold change >1.5; log2 fold change <−0.58, >0.58; *p*-value < 0.05) (**b**). GO terms enriched among genes downregulated or upregulated upon Isl1 loss-of-function and representative genes within these GO terms are presented on the right side of the panel. **c** Venn diagram representing the overlap of genes bound by Isl1 in E8.25-E9 embryos or ESC-derived CPCs (*n* = 2) and differentially expressed in dissected pharyngeal mesoderm/ hearts of E8.75 *Isl1* knockout embryos as well as OFT and RV of E10.5 *Isl1* hypomorphic embryos compared to control embryos (*n* = 4). **d** Isl1-regulated gene network identified using GeneMANIA and Cytoscape. **e**–**g** GO terms enriched in genes bound by Isl1 in ESC-derived CPCs or E8.25-E9 embryos and deregulated in E8.75 *Isl1* knockout embryos (**e**), in E10.5 OFT+RV of *Isl1* hypomorphic embryos (**f**), or in both E8.75 *Isl1* knockout embryos and E10.5 OFT+RV of *Isl1* hypomorphic embryos (**g**). Examples of genes regulated and bound by Isl1 in (**e**–**g**), showing genome tracks of Isl1 ChIP-Seq in CPCs and RNA-Seq reads, are presented on the right side of the panel

To gain insight into the molecular mechanisms underlying Isl1 function in cardiogenesis we performed RNA-Seq from dissected pharyngeal mesoderm and hearts of E8.75 *Isl1* knockout embryos as well as OFT and RV of E10.5 *Isl1* hypomorphic embryos, structures derived from the Isl1^+^ SHF CPCs (Fig. 3a, b; Supplementary information, Fig. S1a–d, Tables S1 and S2). We identified 569 differentially expressed genes in E8.75 *Isl1* knockout embryos and 899 differentially expressed genes in OFT and RV of E10.5 *Isl1* hypomorphic embryos (fold change >1.5; log2 fold change <−0.58, >0.58; *p*-value < 0.05, Supplementary information, Fig. S2a–d). Gene Ontology (GO) analysis revealed over-representation for GO terms linked to cardiac muscle contraction, heart development, atrial septum and OFT morphogenesis in genes downregulated in E8.75 *Isl1* knockout embryos, whereas genes involved in proximal distal pattern formation were overrepresented in upregulated genes (Fig. 3a). At E10.5, in addition to many transcriptional regulators of cardiac morphogenesis, we found significant overrepresentation of genes involved in cell adhesion and differentiation that have been shown to play key role in cardiac looping morphogenesis, cushion formation, neural crest addition, ventricular and OFT septation (Fig. 3b). Interestingly, we identified genes involved in erythroid development and hemogenic lineage specification in genes upregulated upon Isl1 loss (Fig. 3b).

Next, we performed chromatin immunoprecipitation followed by sequencing (ChIP-Seq) to map the genome-wide binding of Isl1 in dissected cardiogenic regions of E8.25-E9 embryos and in ESC-derived CPCs to identify Isl1 primary downstream targets (Supplementary information, Tables S3 and S4). 71% of genes bound by Isl1 in ESC-derived CPCs were also bound in E8.25-E9 embryos (Fig. 3c). Importantly, 75% of genes deregulated in E8.75 Isl1 knockout embryos were bound by Isl1, whereas 67% of genes deregulated in OFT and RV of E10.5 *Isl1* hypomorphic embryos were bound by Isl1  (Fig. 3c). Gene network analysis uncovered distinct groups of Isl1 primary downstream targets (Fig. 3d). These included: *(i)* transcription factors and epigenetic modifiers, such as *Myocd*,^12^ *Mef2c*, *Hand2*,^24^ known Isl1 downstream targets, and other key regulators of cardiac development, such as *Gata4/5*, *Tbx5/20*, *Msx2*, *Hopx*, *Baf60c* (*Smarcd3*); *(ii)* signaling molecules, such as *Fgf10*,^25^ and other key components of the Wnt, Bmp, Notch and Fgf signaling pathways; *(iii)* cardiomyocyte structural genes and genes involved in cardiac contraction, such as *Ttn*, *Ryr2, Mlc1v, Tmod1, Tpm1, Mybpc3*. The observation that Isl1 binds to cardiac structural genes is consistent with the disrupted sarcomerogenesis in *Isl1* hypomorphic embryos but is somewhat surprising because cardiac structural genes are only highly expressed when Isl1 transcription is turned off.

GO analysis after intersection of the RNA-Seq data with ChIP-Seq data revealed overrepresentation of GO terms linked to heart development, cell adhesion and differentiation, atrial septum and outflow tract morphogenesis, as well as cardiac muscle fiber development and cardiac conduction (Fig. 3e–g). This is consistent with the defects observed in *Isl1* hypomorphic embryos. Importantly, we observed enrichment of GO terms involved in response to hypoxia in E8.75 embryos (Fig. 3e) consistent with the critical role of Isl1 in regulating SHF progenitor cell function in response to spatial differences in oxygenation during cardiogenesis.^26^ Expression analysis confirmed the significant downregulation of selected genes involved in OFT development in dissected pharyngeal mesoderm and OFT regions of E8.5 embryos and in dissected OFT and RV of E10.5 *Isl1* hypomorphic embryos (Supplementary information, Fig. S2e, f). Furthermore, cardiomyocyte structural and contraction genes were significantly downregulated in dissected OFT and RV of E10.5 and RV of E13.5 *Isl1* hypomorphic embryos (Supplementary information, Fig. S2f, g). In contrast, no differences were observed in LV of *Isl1* hypomorphic embryos (Supplementary information, Fig. S2h).

**Fig. 4:**
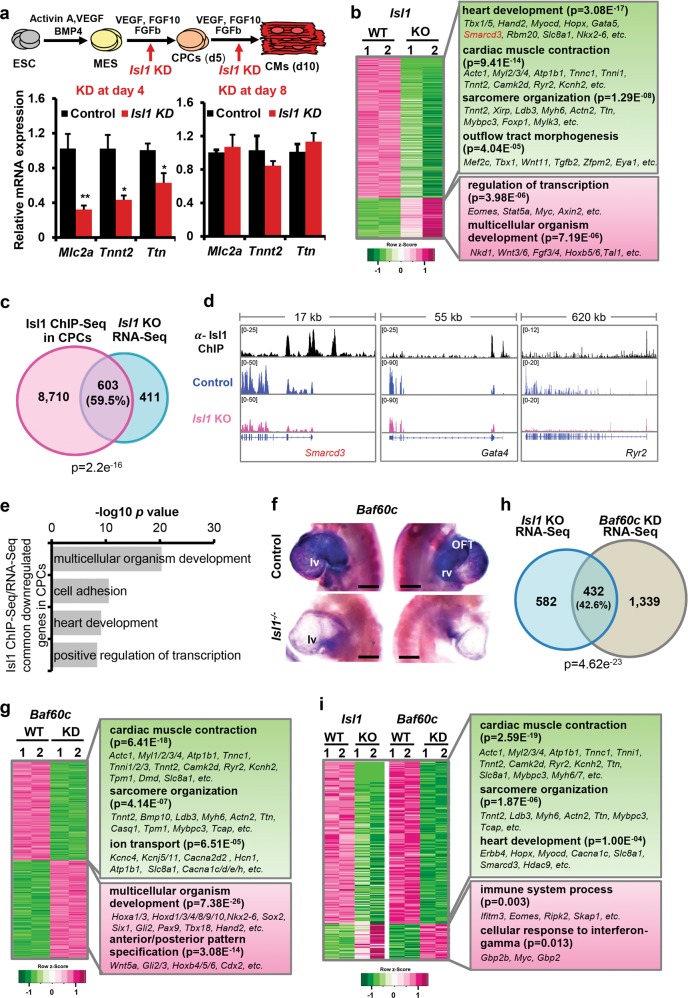
Baf60c is a key Isl1 downstream target. **a** Scheme depicting distinct stages of directed cardiac differentiation (top). Relative mRNA expression of selected cardiomyocyte contraction and structural genes at day 10 of cardiac differentiation after *Isl1* KD at day 4 (left panel) or at day 8 (right panel). Data are presented as mean ± SEM, *n* = 3. **b** Heatmap representation of RNA-Seq analysis of FACS-sorted Nkx2-5^+^ CPCs derived from wild-type and *Isl1*^−/−^ ESCs (*n* = 2; fold change >1.5; log2 fold change <-0.58, >0.58; *p*-value < 0.05). **c** Overlap between genes bound by Isl1 in mESC-derived CPCs and differentially expressed in sorted *Isl1*^−/−^ compared to control CPCs. **d** Examples of genes regulated and bound by Isl1 in mESC-derived CPCs, showing genome tracks of Isl1 ChIP-Seq and RNA-Seq reads of sorted control and *Isl1*^−/−^ mESC-derived CPCs. **e** GO terms enriched in genes bound by Isl1 in mESC-derived CPCs and downregulated in Isl1 knockout CPCs. **f** WT and *Isl1* knockout E9.5 embryos viewed from the left (left panels) and the right (right panels) after in situ hybridization with a Baf60c probe. Scale bars, 200 µm. Abbreviations: rv, right ventricle; lv, left ventricle; OFT, outflow tract. **g** Heatmap representation of RNA-Seq analysis of FACS-sorted Nkx2-5^+^ CPCs derived from control and Baf60c knockdown mESCs (*n* = 2; fold change >1.5; log2 fold change <−0.58, >0.58; *p*-value < 0.05). **h** Overlap of deregulated genes in sorted *Isl1*^−/−^ and Baf60c knockdown CPCs. **i** Heatmap of genes downregulated or upregulated in both *Isl1* knockout and *Baf60c* knockdown versus control CPCs. Representative genes and enriched GO terms are presented on the right side

Isl1 directly bound genes encoding structural and contractile components of cardiomyocytes. This, together with the downregulation of expression of these genes in *Isl1* hypomorphs at later developmental time points when Isl1 is no longer expressed, support the hypothesis that Isl1 might play a decisive role in establishing a transcriptional memory of cardiomyocyte lineage fate commitment during heart development.^27,28^ To further confirm the transient requirement of Isl1 in CPCs for expression of such genes for structural and contractile cardiomyocyte components, we knocked down Isl1 at distinct stages of directed cardiac differentiation of mouse embryonic stem cells (mESCs)^29^ (Fig. 4a; Supplementary information, Fig. S3a, b): at day 4 during the differentiation of mesodermal precursors into cardiac progenitors; or at day 8, after the appearance of beating cardiomyocytes. *Isl1* mRNA levels were high at both stages in control cells (Supplementary information, Fig. S3c). In all our ESC-based experiments we used Nkx2-5–GFP mESCs, which allows monitoring of the differentiation efficiency of ESCs (Supplementary information, Fig. S3d–f). Importantly, cardiomyocyte marker genes and Isl1 direct targets were significantly downregulated at day 10 of cardiomyocyte differentiation only when Isl1 was downregulated at day 4 but not at day 8 (Fig. 4a). Taken together, these data support the requirement of Isl1 in CPCs in setting up a transcriptional program to ensure proper cardiomyocyte differentiation.

### Baf60c is a key Isl1 downstream target

Epigenetic mechanisms are central to establishment and maintenance of transcriptional memory.^28,30–33^ To find epigenetic modifiers employed by Isl1 to establish inheritance of cardiomyocyte lineage identity, and to further identify genes directly regulated by Isl1 in CPCs, we performed RNA-Seq of sorted Nkx2-5–GFP^+^CPCs derived by differentiation of control or *Isl1*^−/−^ Nkx2-5–GFP mESCs (Supplementary information, Fig. S4a, b). We identified 1014 differentially expressed genes (fold change >1.5; log2 fold change <−0.58, >0.58; *p*-value < 0.05, Fig. 4b, Supplementary information, Fig. S4c, d, Table S5). Intersection of this RNA-Seq data to ChIP-Seq data from ESC-derived CPCs revealed 59.5% overlap and identified 603 genes that were bound by Isl1 in CPCs and were deregulated upon Isl1 loss (Fig. 4c). GO analysis of genes downregulated in *Isl1*^−/−^ CPCs that were bound by Isl1 revealed over-representation of GO terms linked to cell adhesion, positive regulation of transcription and heart development (Fig. 4e). In contrast, upregulated genes were enriched for genes involved in hematopoiesis (Supplementary information, Fig. S4e), consistent with the results from *Isl1* loss-of-function embryos. Given the antagonistic relationship between hemangiogenic and cardiogenic mesoderm specification,^34^ this might suggest that Isl1 is important for establishing cardiac fate and prevents the acquisition of hemogenic fate. Comparison of Isl1 primary downstream targets deregulated in E8.75 *Isl1* knockout embryos and *Isl1* knockout ESC-derived CPCs identified known Isl1 downstream targets, such as *Myocd* and *Mef2c*,^24^ as well as many novel primary downstream targets with a role in cardiogenesis (Fig. 4d, Supplementary information, Fig. S4f). One of the targets, *Baf60c* (*Smarcd3*), a cardiac-specific component of the Brg1-based SWI/SNF chromatin remodeling complex plays a crucial role in heart development.^19,20^ *Baf60c* levels were significantly decreased in *Isl1* hypomorphic embryos and *Isl1* knockout embryos, confirming Baf60c as a primary downstream target of Isl1 (Fig. 4f, Supplementary information, Fig. S4f, g). To address whether Baf60c may play a role in mediating Isl1 function in cardiogenesis we performed RNA-Seq of sorted Nkx2-5–GFP^+^CPCs derived by directed differentiation of control or *Baf60c* knockdown Nkx2-5–GFP mESCs.^29^ The RNA-Seq data identified 1771 differentially expressed genes following *Baf60c* depletion (fold change >1.5; log2 fold change <−0.58, >0.58; *p*-value < 0.05, Supplementary information, Fig. S4h, i, Table S6). GO analysis of downregulated genes upon Baf60c depletion showed that Baf60c activates genes involved in cardiac muscle contraction, sarcomere organization and ion transport but represses genes involved in anterior/posterior pattern specification (Fig. 4g). Intersection of the RNA-Seq datasets from *Isl1* KO and *Baf60c* knockdown ESC-derived CPCs showed that 42.6% of the genes deregulated by *Isl1* loss-of-function are also deregulated upon *Baf60c* knockdown (Fig. 4h) and revealed that genes activated by both Isl1 and Baf60c are involved in cardiac muscle contraction and sarcomere organization (Fig. 4i). These results indicate that Baf60c may work in an axis with Isl1 to promote chromatin reorganization at cardiomyocyte structural and contractile protein encoding genes in CPCs well before these genes are highly expressed in differentiating cardiomyocytes.

### Isl1 works in concert with the Brg1-based SWI/SNF complex to regulate its target gene expression

**Fig. 5:**
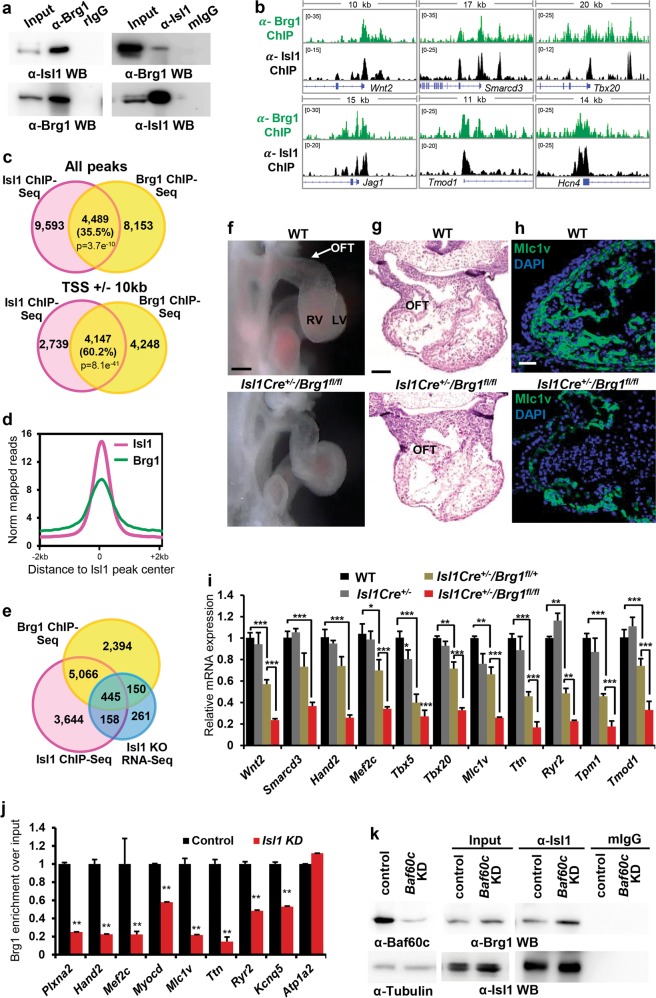
Isl1 works in concert with the Brg1 SWI/SNF complex to regulate its target gene expression. **a** Co-immunoprecipitation showing interaction between Isl1 and Brg1 in E8.25-E9 embryos. **b** Examples of genes bound by Isl1 and Brg1, showing genome tracks of Isl1 ChIP-Seq and Brg1 ChIP-Seq reads. **c** Venn diagram representing the overlap of all Isl1 and Brg1 ChIP-Seq peaks^36^ (top) or of peaks at Transcription Start Sites (TSS)+/− 10 kb (bottom). P value was calculated with Fisher’s exact test. Only high confidence peaks, found in the two ChIP-Seq replicates, were used in the analysis. **d** Average Isl1 and Brg1 ChIP-Seq tag intensities at Isl1 peaks. (**e**) Venn diagram representing the overlap of genes bound by Isl1 and Brg1 (*n* = 2) and differentially expressed in *Isl1*^−/−^ CPCs (*n* = 2). **f** Control and *Isl1-Cre*^*+/−*^*/Brg1*^*fl/fl*^ E9.75 embryos viewed from the right. Scale bars, 500 µm. **g** Histological analyses of control and *Isl1-Cre*^*+/−*^*/Brg1*^*fl/fl*^ E10.5 embryos. Scale bars, 500 µm. **h** Immunostaining of right ventricles of control and *Isl1-Cre*^*+/−*^*/Brg1*^*fl/fl*^ E11.5 hearts for *Mlc1v*. Scale bar, 50 µm. **i** Relative mRNA expression levels of Isl1/Brg1 common targets in dissected OFT and RV of wild-type, *Isl1-Cre*^*+/−*^, *Isl1-Cre*^*+/−*^*/Brg1*^*+/fl*^ and *Isl1-Cre*^*+/−*^*/Brg1*^*fl/fl*^ E10.5 embryos. Data are mean ± SEM, *n* = 4. **j** ChIP-qPCR to analyze Brg1 occupancy at Isl1 bound sequences in control and *Isl1* KD CPCs. *Atp1a2*, a gene not bound by Isl1, serves as a negative control. **k** Co-immunoprecipitation with anti-Isl1 antibody and Western blot analysis for Brg1 in control or *Baf60c* KD CPCs, showing that the interaction between Isl1 and Brg1 does not depend on Baf60c

Baf60c is a cardiac-specific subunit of the Brg1-based SWI/SNF complex, which plays an important role in heart development.^19^ To analyze whether Isl1 may also work in concert with the Brg1-Baf60c based SWI/SNF complex we first analyzed whether Isl1 interacts with Brg1 and Baf60c. Co-immunoprecipitation experiments in ESC-derived CPCs revealed that Isl1 binds to both Brg1 and Baf60c (Supplementary information, Fig. S5a). The binding of Isl1 to Brg1 was further validated in E8.75-E9 embryos (Fig. 5a). Comparison of ChIP-Seq data for Brg1^35,36^ and Isl1 showed significant co-occupancy of Brg1 and Isl1 at Isl1 binding sites (Fig. 5b–d). Furthermore, 59.3% of the genes bound by Isl1 were bound by Brg1, while 44% of the genes deregulated by *Isl1* loss of function were concomitantly bound by Isl1 and Brg1 (Fig. 5e), suggesting that Isl1 may work together with the Brg1-Baf60c complex to regulate nucleosomal structure and expression of its targets. To investigate this hypothesis in vivo, we inactivated *Brg1*^37^ in SHF progenitor cells using an *Isl1*-Cre driver line. *Brg1*-deficient embryos showed a shortened outflow tract, a small right ventricle at E9.5 and died by E14.5 (Fig. 5f–h). Immunohistochemical stainings and qPCR expression analysis of dissected OFT and RV of E10.5 wild-type, *Isl1-Cre*^*+/−*^, *Isl1-Cre*^*+/−*^*/Brg1*^*fl/+*^ and *Isl1-Cre*^*+/−*^*/Brg1*^*fl/fl*^ embryos revealed significant downregulation of Isl1 primary targets in *Isl1-Cre*^*+/−*^*/Brg1*^*fl/+*^ embryos and further decrease in Brg1-deficient embryos (Fig. 5h, i), whereas no major changes were observed in the left ventricles of these embryos (Supplementary information, Fig. S5b) or in the expression of genes, that are not Isl1 targets, in the right ventricle (Supplementary information, Fig. S5c). The dosage-sensitive interdependence of Isl1 and Brg1 supports the hypothesis that the Brg1-Baf60c complex might regulate gene expression in concert with Isl1. We next examined whether Brg1 was directed to Isl1 target sequences via Isl1. Knockdown of *Isl1* led to a significant reduction of Brg1 occupancy at Isl1 targets (Fig. 5j), suggesting that Isl1 might recruit the Brg1 complex to promote chromatin reorganization at its target genes.

Baf60c mediates interactions between cardiac transcription factors such as Tbx5, Nkx2-5, Gata4 and the Brg1 complex to drive cardiac specific gene expression.^19–21^ To test whether Baf60c promotes the binding of Isl1 to the Brg1 complex, we performed co-immunoprecipitation in control and *Baf60* KD ESC-derived CPCs using Isl1 antibody. Similar binding of Isl1 and Brg1 was observed in control and Baf60-depleted CPCs suggesting that, in contrast to Tbx5, Nkx2-5 and Gata4, Baf60c does not mediate the association of Isl1 with the Brg1 complex (Fig. 5k). Consistent with a role of Isl1 in not only regulating Baf60c levels but also working together with the Brg1-Baf60c complex, overexpression of Baf60c was not sufficient to rescue the cardiac differentiation defect of *Isl1* knockdown ESC and the expression of Isl1-Baf60c target genes (Supplementary information, Fig. S5d, e).

### Isl1 acts as a pioneer factor in cardiogenesis

**Fig. 6:**
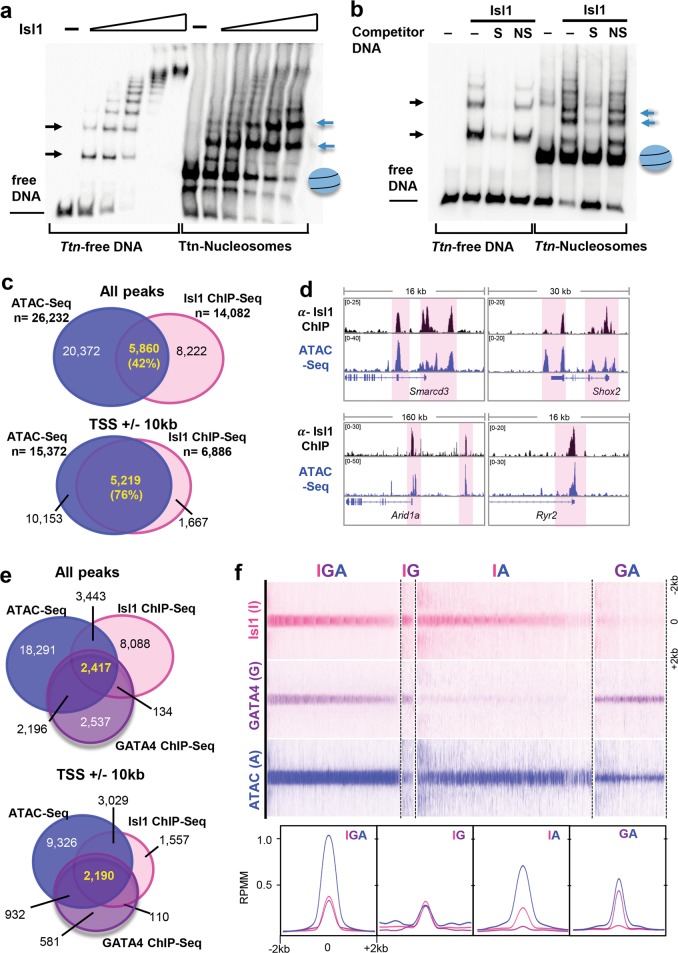
Isl1 recognizes its DNA binding motif on DNA wrapped around nucleosomes and its binding correlates with sites of open chromatin. **a** EMSA with increasing amount of recombinant Isl1 protein (0, 0.2, 0.4, 0.6, 0.8 and 1 µM) and free *Ttn* DNA fragment harbouring Isl1 binding sites (left panels) and the same fragment assembled into nucleosomes (right panels). **b** Competition assay showing the specificity of Isl1 binding to free and nucleosomal *Ttn* DNA using specific Isl1 binding oligo and nonspecific oligo as competitors at 80× molar ratio excess. S, specific competitor; NS, nonspecific competitor. **c** Overlap of all Isl1 ChIP-Seq and ATAC-Seq peaks (top; *n* = 2, each) or peaks at Transcription Start Sites (TSS)±10 kb in mESC-derived CPCs. Only high confidence peaks, found in two Isl1 ChIP-Seq and ATAC-Seq replicates, were used in the analysis. **d** Examples of genes bound by Isl1, showing open chromatin configuration at Isl1 binding sites in mESC-derived CPCs. Genome tracks of Isl1 ChIP-Seq and ATAC-Seq reads of mESC-derived CPCs. **e** Overlap of all Isl1 ChIP-Seq (*n* = 2), GATA4 ChIP-exo^5^ (*n* = 3) and ATAC-Seq peaks (*n* = 2) (top) or peaks at Transcription Start Sites (TSS)±10 kb in CPCs. **f** Heatmap (top) and aggregation plot (bottom) of mapped reads of Isl1 ChIP-Seq, GATA4 ChIP-exo and ATAC-Seq at  2 kb around peak midpoints of Isl1, GATA4 and ATAC (IGA); Isl1 and GATA4 (IG); Isl1 and ATAC (IA); GATA4 and ATAC (GA) occupancy groups

Pioneer transcription factors play critical roles in programming the epigenome and instructing lineage specification and differentiation.^1,2,4^ To initiate cell programming pioneer transcription factors engage “closed” chromatin covered by nucleosomes. To test whether Isl1 could bind to DNA wrapped around nucleosomes we performed in vitro electrophoretic mobility shift assays (EMSAs). For the analysis we selected a DNA probe containing Isl1 motifs within the *Ttn* promoter region, which was bound by Isl1,^38^ and downregulated in Isl1 and Baf60c depleted CPCs. As expected, recombinant Isl1 (Supplementary information, Fig. S6a) bound to the free DNA probe within *Ttn* promoter region (Fig. 6a, lanes 1-6). Next, we assembled the *Ttn*-DNA into nucleosomes by salt gradient dilution assembly with recombinant human histones (Supplementary information, Fig. S6b, c). Importantly, Isl1 showed similar binding to DNA assembled in nucleosomes compared to free DNA (Fig. 6b, lanes 7–12). Competition assays using a 80-fold molar excess of specific competitor DNA probe containing Isl1 binding sites, but not probe containing non-specific sequences, supported the specific binding of Isl1 to free *Ttn*-DNA and *Ttn*-DNA assembled into nucleosomes (Fig. 6b). In contrast, Nkx2.5 bound free *Ttn* DNA but was not able to bind *Ttn*-DNA assembled into nucleosomes (Supplementary information, Fig. S6d).

Pioneer factor binding imparts competence for transcription by chromatin opening. To further test whether Isl1 acts as a pioneer factor, we analyzed whether Isl1 binding might induce the formation of accessible chromatin in CPCs by performing genome-wide analysis of open chromatin landscapes using ATAC (‘assay for transposase-accessible chromatin’) sequencing (ATAC-Seq)^39^ in mESC-derived CPCs (Supplementary information, Table S7). Comparison of ATAC-Seq data and Isl1 binding profiles revealed a 42% overlap of Isl1 ChIP-Seq with ATAC-Seq peaks in CPCs. At the promoter proximal regions (TSS±10 kb) 76% of Isl1 binding sites showed open chromatin (Fig. 6c, d), suggesting a role of Isl1 binding in the formation of accessible chromatin required for cardiogenesis, similar to other “pioneer factors”. The core cardiac transcription factor GATA4 represents a prototypical example of a pioneer factor, it binds efficiently to its target sequences on nucleosomal DNA^40^ and induces chromatin reorganization driving heart development and disease.^41^ To address whether GATA4 binding might affect Isl1 binding or vice versa, we first compared GATA4^5^ and Isl1 binding sites in CPCs with ATAC-Seq peaks. Using the published GATA4 ChIP-exo average footprints^5^ (footprints present in at least two replicates), we observed 232 sites that were bound by GATA4 and Isl1 that showed accessible chromatin, whereas 5628 Isl1 ChIP-Seq peaks overlapped with ATAC-Seq peaks (Supplementary information, Fig. S6e). Heatmap analysis, however, showed relatively high GATA4 signal at Isl1-ATAC only sites, suggesting dynamic GATA4 binding (Supplementary information, Fig. S6f). Therefore, we called peaks in each GATA4 ChIP-exo replicate and used high confidence peaks found in all three ChIP-exo replicates for further analysis. This analysis identified 2417 common Isl1-GATA4-ATAC peaks, showing that Isl1 and GATA4 often co-occupy sites characterized with open chromatin (Fig. 6e, f). In addition, we identified accessible chromatin sites bound only by Isl1 or GATA4, supporting the notion that Isl1, similar to GATA4, functions as a pioneer transcription factor (Fig. 6e, f).

**Fig. 7:**
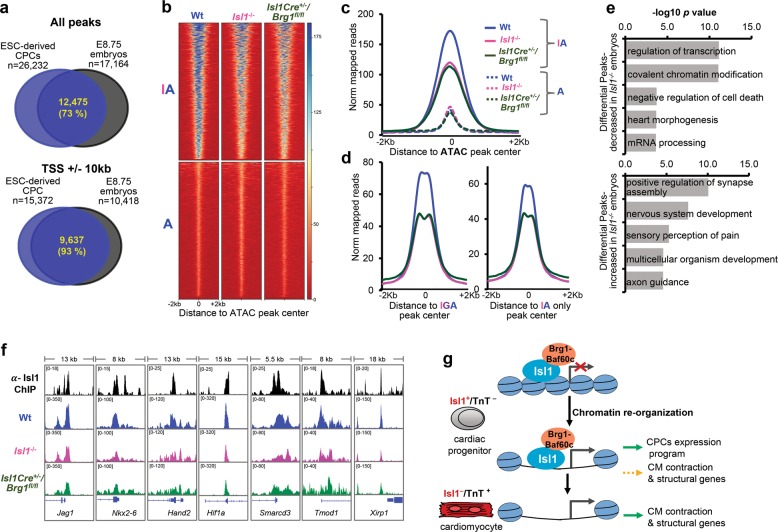
Isl1 and the Brg1-based SWI/SNF complex shape the chromatin landscape at Isl1 targets in CPCs. **a** Overlap of all ATAC-Seq peaks in ESC-derived CPCs (*n* = 2) and pharyngeal mesoderm/hearts of E8.75 embryos (*n* = 3) (top) or peaks at Transcription Start Sites (TSS)±10 kb (bottom). Only high confidence peaks, found in the two ATAC-Seq replicates of ESC-derived CPCs or in the three ATAC-Seq triplicates of E8.75 embryos, were used in the analysis. **b** Heatmap of tag densities of ATAC-Seq signal in wild-type, *Isl1*^*−/−*^ and *Isl1-Cre*^*+/−*^*Brg1*^*fl/fl*^ embryos at top 1000 Isl1-ATAC (IA) or ATAC only (A) peaks. **c** Average ATAC-Seq tag intensities in wild-type, *Isl1*^*−/−*^ and *Isl1-Cre*^*+/−*^*Brg1*^*fl/fl*^ embryos at Isl1-ATAC (IA) or ATAC only (A) peaks. **d** Average ATAC-Seq tag intensities at common peaks between Isl1, GATA4 and ATAC-Seq in wild-type, *Isl1*^*−/−*^ and *Isl1-Cre*^*+/−*^*Brg1*^*fl/fl*^ embryos (left) or at Isl1 and ATAC-Seq only peaks. **e** GO terms enriched in ATAC-Seq peaks decreased (top panel) or increased (lower panel) more than 2 fold (*p* < 0.05) in Isl1^*−/−*^ versus control embryos. **f** Examples of genes showing decreased ATAC-Seq signal at Isl1 binding sites. Genome tracks of Isl1 ChIP-Seq and ATAC-Seq of wild-type, *Isl1*^*−/−*^ and *Isl1-Cre*^*+/−*^*Brg1*^*fl/fl*^ embryos are presented. **g** Model of the role of Isl1 in cardiogenesis by controlling epigenetic mechanisms and memory. Isl1 binds to compacted chromatin at genes involved in CPC function, as well as to genes involved in cardiomyocyte contraction and structural organization. Isl1 works in concert with the Brg1-Baf60c-based SWI/SNF complex to open up chromatin and allow gene expression directly in CPCs, or at a later time point in cardiomyocytes

Finally, we analyzed whether loss of Isl1 or Brg1 might affect chromatin opening by performing ATAC-Seq in E8.75 (6 somites) wild-type, *Isl1*^*−/−*^ or *Isl1-Cre*^*+/−*^*/Brg1*^*fl/fl*^ embryos (Supplementary information, Table S8). We observed 73% overlap of all ATAC-Seq peaks in pharyngeal mesoderm and hearts of E8.75 embryos compared to ESC-derived CPCs (Fig. 7a) whereas we observed 93% overlap at promoter proximal regions (TSS±10 kb, Fig. 7a) showing high similarity between the open chromatin of in vitro differentiated CPCs to CPCs in early embryos. Importantly, we observed significant reduction of chromatin accessibility at Isl1-ATAC peaks (i.e. sites bound by Isl1 showing open chromatin conformation) upon *Isl1* and *Brg1* loss (Fig. 7b, c), whereas chromatin accessibility was not affected at ATAC only sites (not bound by Isl1). Reduction in chromatin accessibility was observed in both Isl1-ATAC sites bound or not bound by GATA4 (IGA or IA only), supporting the notion that Isl1 acts as pioneer factor also independently of GATA4 (Fig. 7d). In addition, GATA4 binding was decreased in Isl1 depleted CPCs (Supplementary information, Fig. S7a). However, we cannot pinpoint whether Isl1 is necessary for GATA4 association at Isl1 target sites or whether lower GATA4 occupancy could be due to the decreased levels of GATA4 in Isl1-deficient CPCs (Fig. 4d, Supplementary information, Table S5). In addition, the high average ATAC signal at Isl1-ATAC sites compared to ATAC only sites further supports a role of Isl1 in chromatin opening in CPCs (Fig. 7c). GO analysis of differential ATAC-Seq peaks in *Isl1* and/or *Brg1*-deficient embryos revealed that the Isl1/Brg1 complex plays a key role in activating the expression program essential for heart development while repressing nervous system development and cell adhesion (Fig. 7e, Supplementary information, Fig. S7b). Importantly, we observed significant overrepresentation of GO terms related to chromatin modification in ATAC-Seq peaks decreased in Isl1 knockout embryos, including multiple components of the SWI/SNF chromatin remodeling complexes such as Arid1a (Baf250), Pbrm1 (Baf180); Dpf3 (Baf45c), Smarcd3 (Baf60c) as well as Brg1/Smarca4 (Fig. 7f, Supplementary information, Fig. S7c, Table S7). Genes showing decreased chromatin accessibility only in Isl1 knockout embryos mostly encoded factors involved in transcriptional regulation, suggesting that Isl1 might work together with other chromatin modifiers to regulate their expression (Supplementary information, Fig. S7c). Genes not bound by Isl1 did not show changes in chromatin accessibility (Supplementary information, Fig. S7c). In a complementary approach, we observed an increase of H3 occupancy at Isl1/Brg1-Baf60c targets in Isl1 and Baf60c knockdown CPCs compared to control CPCs (Supplementary information, Fig. S7d). Isl1-positive cardiovascular progenitors are multipotent and can differentiate into all three cardiovascular lineages: cardiomyocytes, smooth muscle cells and endothelial cells.^14^ Analysis of chromatin accessibility at promoters of cardiac, smooth muscle and endothelial development/differentiation genes revealed a decrease in open chromatin at all cardiovascular lineage-related genes in *Isl1* and *Brg1*-deficient embryos (Supplementary information, Fig. S8). Notably the ATAC-Seq signal was highly decreased at genes involved in cardiac contraction. Nevertheless, these data suggest that Isl1 might play a pioneering function not only for cardiomyocytes but also for other cardiovascular lineages and that it requires the Brg1-based SWI/SNF chromatin remodeling complex.

Taken together these data suggest a pioneering function of Isl1 by binding to inaccessible chromatin and working in concert with the Brg1-Baf60c-based SWI/SNF complex to confer permissive lineage-specific alterations in CPC chromatin landscape. These allow gene expression directly in CPCs or at a later time point in cardiomyocytes (Fig. 7g).

## Discussion

Pioneer transcription factors play critical roles in programming the epigenome during lineage specification.^1–4^ Our study shows that Isl1 acts as a pioneer transcription factor in heart development by shaping the chromatin landscape in CPCs and orchestrating a complex gene regulatory network driving cardiac development and defining cardiomyocyte identity. Similar to other well-characterized pioneer factors, such as forkhead box A (FOXA) factors, GATA-binding (GATA) factors, PU.1^4^ and the pluripotency factors OCT4, SOX2 and KLF4,^1^ Isl1 recognizes its DNA binding motif even when the DNA is wrapped around nucleosomes, which enables it to engage its target sites even in condensed chromatin. ATAC-Seq of ESC-derived CPCs and CPCs in embryos revealed that open chromatin regions are centered on Isl1 binding sites and loss of Isl1 led to significant decrease of chromatin accessibility, suggesting an important role of Isl1 binding in the formation of accessible chromatin. We further show that Isl1 works in concert with the Brg1-based SWI/SNF complex to promote chromatin accessibility and establish competence for lineage specific gene activation.

Using an *Isl1* hypomorphic mouse line, genome-wide profiling of Isl1 binding together with RNA- and ATAC-sequencing of cardiac progenitor cells and their derivatives, we uncover a regulatory network downstream of Isl1 that orchestrates cardiogenesis (Fig. 3d). We show that Isl1 binds to and regulates the expression of transcription factors, epigenetic modifiers and signaling molecules with critical functions and high expression in SHF CPCs.^8,10^ An important group of Isl1 target genes play key roles in development of the atrioventricular canal and in outflow tract morphogenesis, including *Wnt2/5/11*, *Tgfb2*, *Jag1*, *Maml1*, *Smad6*, *Pbx3*, *Msx2*, *Fog2* (*Zfpm2*), *Tbx3*/*5*/*20*, and *Plxna2*.^42^ Consistent with this we observed various degrees of cardiac outflow tract septation abnormalities, including partial outflow tract septation and misalignment or common arterial trunk, ventricular septal defects and atrial septal defects in *Isl1* hypomorphic mouse embryos. Similarly, genetic variations in *ISL1* have been associated with susceptibility to ventricular septal defect^43^ and non-syndromic, complex congenital heart disease^17^ in human patients, whereas *ISL1* haploinsufficiency is associated with d-transposition of the great arteries.^16^ Thus, our *Isl1* hypomorphic mouse model represents a valuable genetic system to gain new insights into the etiology of congenital heart defects and for developing novel therapeutic strategies.

While Isl1 binding to genes with critical functions and high expression in SHF CPCs might have been expected, it was surprising to see that Isl1 binds to cardiomyocyte structural genes and genes involved in cardiomyocyte function in CPCs well before these genes are highly expressed in differentiating cardiomyocytes. Consistent with this, loss of Isl1 was associated with significantly reduced expression of cardiomyocyte structural genes and genes involved in cardiac contraction and sarcomerogenesis that were bound by Isl1 in CPCs. This suggests that Isl1 directly controls cardiomyocyte identity, CPC differentiation and sarcomeric maturation. We reason that Isl1 binds to closed chromatin and spawns permissive cardiomyocyte lineage-specific alterations in the chromatin landscape of CPCs, which enables subsequent recruitment of additional regulatory factors activating these genes in cardiomyocytes when Isl1 itself is switched off. Thus, this establishes a lasting regulatory network driving cardiogenesis. We did not observe any change in the expression of Isl1 primary target genes in the left ventricle of *Isl1* hypomorphic embryos whereas they were significantly changed in dissected right ventricles, suggesting a specific role of Isl1 in the second heart field. However, we found a strong decrease of *Baf60c* in the left ventricle of *Isl1* knockout embryos. In addition, other studies have also observed downregulation of Isl1 targets in the whole hearts of *Isl1* knockouts.^9,12^ Since Isl1 has been reported to be transiently expressed in CPCs of the first heart field, we cannot exclude a possibility that Isl1 plays a pioneer role in the first heart field. In addition, analysis of chromatin accessibility at promoters of cardiac, smooth muscle and endothelial development/differentiation genes revealed a decrease in open chromatin at all cardiovascular lineage-related genes in *Isl1* and *Brg1*-deficient embryos, suggesting that Isl1 may also play a pioneering role for cardiac endothelial cell lineage settlement. This is consistent with single cell analyses demonstrating the critical role of Isl1 in cardiovascular progenitor fate bifurcation into the endothelial and cardiomyocyte lineages.^44^

We demonstrate that Isl1 works together with the Brg1-Baf60c complex to induce chromatin reorganization at its target sites and promote cardiac differentiation. Consistent with this, depletion of either *Isl1*, *Brg1* or *Baf60c* led to a significant decrease of chromatin accessibility at Isl1 target genes and depletion of *Brg1* in Isl1^+^CPCs led to defects in cardiac morphogenesis, cardiomyocyte differentiation and the expression of Isl1 primary downstream targets. The key importance of the Brg1-based SWI/SNF complex in heart development is also evidenced by genetic studies in mice, showing that Brg1 haploinsufficiency leads to various cardiac morphogenetic defects.^19^ A large body of studies has demonstrated that the SWI/SNF complexes undergo progressive changes in subunit composition during developmental transitions and that the unique subunit composition at each developmental stage correlates with a gene expression program that is required for maintaining a particular cell state.^45–47^ Similarly, the inclusion of distinct BAF subunits promotes temporally distinct gene expression programs in cardiogenesis.^36^ Interestingly, we found a significant decrease of chromatin accessibility at multiple components of the SWI/SNF chromatin remodeling complexes such as *Arid1a* (*Baf250*), *Dpf3* (*Baf45c*), *Smarcd3* (*Baf60c*) as well as *Brg1* (*Smarca4*) in *Isl1* knockouts and decreased expression in CPCs or *Isl1* hypomorphic and *Isl1* knockout embryos, suggesting a mechanism by which Isl1 reinforces its function in chromatin reorganization to drive cardiogenesis. Ablation of BAF250a, a critical regulatory subunit in the SWI/SNF BAF complex, in the SHF using *Mef2c-Cre* leads to persistent truncus arteriosus, trabeculation defects, reduced cardiomyocyte proliferation and differentiation, and embryonic lethality around E13, similar to our data from ablation of Brg1 with *Isl1-Cre*. The cardiac-specific subunits of the complex, Baf45c and Baf60c, also play instrumental roles during cardiogenesis and cardiomyocyte differentiation.^20,48,49^ The Brg1-Baf60c activity was shown to direct ectopic differentiation of mouse non-cardiogenic mesoderm into beating cardiomyocytes in concert with Gata4 and Tbx5 by permitting the binding of Gata4 to cardiac specific genes.^21^ We found that Baf60c together with Isl1 is required for the activation of genes involved in cardiomyocyte contraction, sarcomere organization and transcriptional regulation, whereas Baf60c alone activates genes participating in ion transport. This is consistent with the gene expression changes observed in *Baf60c* knockout embryos.^49^ During cardiogenesis, Baf60c mediates interactions between the core cardiac transcription factors Tbx5, Nkx2-5, Gata4 and the Brg1 complex.^19–21^ In contrast, Baf60c does not seem to moderate Isl1 binding to the Brg1 complex. However, the loss of *Baf60c* upon *Isl1* depletion may additionally affect the function of Tbx5, Nkx2-5 and Gata4, as an imbalance between their levels and the Brg1-Baf60c complex was shown to result in impaired cardiac development and transcriptional activation of their targets.^19^

Given that a set of transcription factors can induce direct or partial reprogramming via CPCs of non-myocyte cells into functional cardiomyocytes,^50–52^ it is of utmost interest to find the optimal set of factors and understand how their activities could be enhanced to improve cell reprogramming for clinical applications. Although many cocktails of regulatory factors have been employed in direct cardiac reprogramming, the resultant cells often are immature and/or incompletely specified and the efficiency is low. It has been suggested that a transition through an early lineage intermediate, in which pioneer transcription factors of cell fate can engage naïve chromatin to induce hierarchical lineage specific regulatory networks, may improve the efficiency of reprogramming.^53^ The discovery of Isl1 as a pioneering factor in cardiogenesis can help to develop more efficient strategies for cardiac reprogramming.

In conclusion, our study highlights a pioneering function of Isl1 in cardiac lineage commitment and provides exciting novel insights into the molecular machinery orchestrating cardiogenesis.

## MATERIALS AND METHODS

All animal experiments were done in accordance with the Guide for the Care and Use of Laboratory Animals published by the US National Institutes of Health (NIH Publication No. 85-23, revised 1996) and according to the regulations issued by the Committee for Animal Rights Protection of the State of Hessen (Regierungspraesidium Darmstadt) and of the Tongji University School of Medicine (TJmed-010-10).

### Histology

Embryos were sacrificed by cervical dislocation and hearts were isolated and fixed with 4% paraformaldehyde overnight at 4 °C. After fixation the hearts were dehydrated in ethanol and stored at −20 °C for further paraffin embedding, sectioning and H&E staining. For histological analysis hearts were incubated in xylol and embedded in paraffin. Hematoxylin and eosin staining was performed according to the manufacturer’s instruction (Sigma, GHS116, HT-110216). Representative images of histological analysis of minimum six embryos with the same genotype are presented.

### Magnetic resonance imaging (MRI)

Embryos were analyzed by MRI, as previously described.^54^ Briefly, paraformaldehyde-fixed embryos were embedded in a MRI contrast agent and imaged using an 11.7 Tesla magnet. The 3D MRI dataset obtained was reconstructed into axial TIFF slices. Image datasets were analyzed and 3D reconstructions created using Amira 3.0 software.

### Immunofluorescence and imaging

Paraffin sections were de-paraffinized and re-hydrated. Heat-induced epitope retrieval was performed in 10 mM sodium citrate, pH 6.0. Sections were blocked for 1 h (10% fetal bovine serum in 1× PBS+0.5% Triton X-100) followed by incubation with anti-myosin heavy chain antibody 1:50 dilution (MF20, DSHB) and Mlc1v (Santa Cruz, SC-47719) overnight at room temperature. Sections were washed three times in 1× PBS/0.5% Triton X-100 for 10 min and incubated with respective secondary antibodies, e.g., anti-mouse Alexa 594 (Thermo Fisher Scientific, A11005), anti-rabbit Alexa 488 (Thermo Fisher Scientific, A21206) along with DAPI for 1 h at room temperature. 50% glycerol was used for mounting and image acquisition was done using a Zeiss LSM 710 confocal microscope. Representative images of immunostainings of three embryos with the same genotype are presented.

### ES Cell culture and differentiation

E14 Tg(Nkx2–5-EmGFP) ES cells^55^ were maintained on mitomycin treated mouse embryonic fibroblasts (MEF) in Knockout DMEM medium (GIBCO, 10829018) containing 4.5 mg/ml D-glucose, supplemented with 10% serum replacement (GIBCO, 10828028), 2 mM L-Glutamine, 0.1 mM 2-mercaptoethanol (Sigma, M3148), 1 mM sodium pyruvate (GIBCO, 11360039), and 1,000 U/ml of leukemia inhibitory factor (ESG1107, Millipore). ES cells were differentiated in cardiomyocytes either by the standard hanging drop method^56^ or by directed differentiation of ES cells into cardiomyocytes according to the protocol described in.^29^ Briefly, for directed cardiomyocyte differentiation ES cells were grown on gelatin in Neurobasal medium: DMEM/F12 (1:1; GIBCO, 21103049 and 21331020) medium supplemented with 2000U/ml LIF and 10 ng/ml BMP4 (R&D, 314-BP) for 2 days and differentiated by aggregation in low attachment bacterial dishes at a cell density of 75000 cells/ml in IMDM: F12 medium (3:1; GIBCO, 12440053 and 11765054). After 48 h aggregates were dissociated and re-aggregated in the presence of Activin A (R&D, 338-AC; 5 ng/ml), VEGF (R&D, 293-VE-010; 5 ng/ml) and BMP4 (R&D, 314-BP; 0.1-0.8 ng/ml; Bmp4 concentration was empirically determined depending on lot). 40 h following the second aggregation, aggregates were dissociated and plated as a monolayer in Stempro-34 medium supplemented with Stempro34 nutrient supplement (GIBCO, 10639011), L-Ascorbic Acid (A4403 Sigma) and VEGF (R&D #293-VE-010; 5 ng/ml), bFGF (R&D, 233-FB; 10 ng/ml), and FGF10 (R&D, 345-FG; 25 ng/ml) growth factors. We usually obtained around 60-80% of Nkx2.5-GFP^+^CPCs using this protocol. Nkx2.5-GFP positive cells were FACS sorted at day 5 CPC stage for RNA extraction.

ES cell differentiation by the standard hanging drop method was induced by dissociating ES cells and aggregating 500 cells in 15 µl drops in ES cell growth medium without LIF. After 48 h in hanging drops the resulting EBs were transferred to low attachment culture dishes. At day 5, EBs were dissociated and CPCs were FACS sorted for GFP expression.

For stable transfections HEK293T cells were seeded on a 6-well plate and were transfected at 50%–80% confluency with 3 µg of plasmids containing shRNA for *Baf60c* (*Sh1*: CGCCTAAAGTTCTCTGAGATT, *Sh2*: GCTGCGCCTTTATATCTCCAA), *Isl1* (CGGCAATCAAATTCACGACCA) or control (pLKO) along with packaging and envelope plasmids obtained from the RNAi consortium (TRC) shRNA library using using X-tremeGENE DNA transfection reagent (Roche, 6366236001). 48 h after transfection the viral supernatant was collected and used to transduce 100.000 ES cells on 5% poly-HEMA (Sigma, P3932) treated plates for 12 h. The following day, the transduced ES cells were plated on feeders and 24 h after transduction were selected with 10 ng/mL puromycin for 2 passages.

For generation of *Isl1*^−/−^ ESCs by CRISPR/Cas9-mediated gene targeting, a combination of two guide RNAs (gRNAs) was used as follows: gRNA-1: 5′- CCGATTTAAGCCGGCGGAGT -3′ and gRNA-2: 5′- TCATGAGCGCATCTGGCCGA -3′. pSpCas9(BB)-2A-Puro (PX459) V2.0, kind gift from Feng Zhang (Addgene plasmid # 62988,^57^) was digested and ligated with annealed gRNAs into the recombinant plasmid pSpCas9(BB)-2A-Puro-gRNA-1 and pSpCas9(BB)-2A-Puro-gRNA-2. Both plasmids were transfected into ESCs using Lipofectamine 2000 according to the manufacturer’s protocol. Positive cells were selected after 24 h for 48 h using puromycin (4 µg/ml) and 5, 000 cells were further plated in a 6-cm dish. After 7 days of culture, single clones were picked and screened by PCR.

### RNA isolation and qRT-PCR analysis

RNA was isolated using TRIzol (Invitrogen, 15596026) or RNeasy mini kit (QIAGEN, 73304) following the manufacturer’s instructions and cDNA synthesis was performed using High-capacity cDNA reverse transcription kit and random primers (Applied Biosystems, 4368813). *Gapdh* or *Actc1* (where specified in the figure legends) were used for normalizing gene expression.

### In situ hybridization

Embryos were fixed overnight at 4 °C in 4% PFA followed by dehydration in methanol. For in situ analysis, embryos were rehydrated and treated with proteinase K (10 µg/ml) for 12 min and re-fixed in 4% PFA, followed by incubation overnight at 65°C with DIG-labeled Baf60c RNA probe in hybridization buffer containing 50% formamide, 5 x SSC, 1% SDS, 5 µg/µl yeast tRNA and 10 µg/µl heparin. Next, the embryos were washed two times with 50% formamide, 5 x SSC, 1%SDS followed by subsequent washes in 2 × SSC and 0.2 x SSC at 65 °C and 1xMABT (100 mM maleic acid, 150 mM NaCl). Embryos were treated with 2× blocking reagent (Roche, 11096176001) for 2 h at room temperature followed by incubation with alkaline phosphatase conjugated anti-DIG antibody (Roche, 11093274910) for 18 h at 4 °C. For color development BM-purple reagent (Roche, 11442074001) was used. Representative image of three control and three *Isl1* knockout embryos were presented.

### Co-immunoprecipitation

For co-immunoprecipitation cardiac progenitor cells differentiated from mouse ES cells and 30 mouse embryos (E8.25-E9) homogenized with plastic pestle were lysed with lysis buffer (50 mM Tris pH 7.5, 10 mM EGTA, 100 mM NaCl, 0.1% Triton X-100) plus protease and phosphatase inhibitors on ice. After sonication and centrifugation, the supernatant was used for immunoprecipitation. Embryoid bodies were trypsinized to obtain a single cell suspension. Nuclei were isolated by incubating in lysis buffer containing 50 mM Tris pH 8.0, 2 mM EDTA pH 8.0, 0.1% NP-40, 10% glycerol along with protease and phosphatase inhibitors. Isolated nuclei were disrupted by sonication in 50 mM Tris pH 8.0, 0.1% SDS, 5 mM EDTA pH 8.0 buffer along with protease and phosphatase inhibitors. 500 µg of protein lysates were precleared with Protein G-Sepharose beads (GE Healthcare, 17-0618) at 4 °C on a rotary shaker for 1 h. Precleared lysates were incubated with 1 µg of α-Isl1 antibody (39.4D5 DSHB) overnight at 4 °C. Protein complexes were bound to Protein G-Sepharose beads for 1 h at 4°C. Protein complex bound beads were washed and resolved in a standard SDS-PAGE system. Brg1 (1:500, Santa Cruz, SC-10768), Isl1 (1:10 dilution; 39.4D5 DSHB) and Baf60c antibody (1 to 500 dilution; a kind gift from Pier Lorenzo Puri) were used for western blotting.

### RNA-Sequencing

Pharyngeal mesoderm/ hearts of E8.75 (6 somites) and OFT+RV of E10.5 control and *Isl1* KO or hypomorphic embryos were dissected and stored at −80 °C in Qiazol. Total RNA from single embryos was extracted using Rneasy Microarray Tissue Kit (Qiagen #73304). Control, *Baf60c* KD and *Isl1* KO Nkx2.5-GFP ES cell lines were differentiated into cardiac progenitors. Nkx2.5-GFP positive cells from two different control and *Isl1*^−/−^ clones or control and Baf60 KD ESCs pools were sorted and RNA was isolated using RNeasy microarray kit (Qiagen #73304). The integrity of RNA was assessed on Bioanalyzer 2100 (Agilent). For RNA-Seq of control, *Isl1* hypomorphic and *Isl1*^−/−^ tissue samples, 100 ng of total RNAs was used for library preparation and sequenced on BGISEQ-500 or on NextSeq500 (Illumina), respectively. For RNA-Seq of control, *Isl1*^−/−^ and *Baf60c* KD CPCs, 1 µg RNA was used as input for Truseq Stranded mRNA library preparation (Illumina). Sequencing was performed on NextSeq500 (Illumina) using V2 chemistry.

### RNA-Seq data analysis

RNA-Seq reads were mapped to the mm9 reference genome using STAR^58^ (−alignIntronMin 20 −alignIntronMax 500000). Samples were quantified using analyzeRepeats.pl^59^ (mm9 –count exons –strand both –noadj). Differential expression (fold change >1.5; log2 fold change <−0.58, >0.58; *p*-value < 0.05) was quantified and normalized using DESEq2. Reads per kilobase per millions mapped (rpkm) was determined using rpkm.default from EdgeR. The KNIME 2.9.1 platform (Konstanz, Germany) was used to filter differentially regulated genes. Gene ontology analysis was performed using DAVID Bioinformatics Resources 6.8. Heatmaps from the RNA-Seq were created using heatmap.2 from ggplot2 in R. Values in the heatmaps represent Z-score calculated from (Log2RPKM+1) after row normalization. The PCA plots in each RNA-Seq dataset were obtained using rowVars and prcomp into a custom R-script.

### Chromatin immunoprecipitation (ChIP) and ChIP sequencing

Mouse embryos at E8.25-E9 were collected into cold PBS. Cardiogenic mesoderm and heart region was crosslinked with 1% formaldehyde at room temperature for 15 min, whereas mESC-derived CPCs for 10 min. Crosslinking was terminated by adding 125 mM glycine followed by three washes with cold PBS. Single cells were obtained by disruption of the tissue using pestle and mortar and nuclei were isolated by incubation in lysis buffer (50 mM Tris pH 8.0, 2 mM EDTA pH 8.0, 0.1% NP-40, 10% glycerol) along with protease and phosphatase inhibitors. Nuclei were frozen at −80 °C until further processing. Around 60-80 E8.25 to E9.0 embryos were pooled and lysed in nuclear lysis buffer supplemented with protease and phosphatase inhibitors (50 mM Tris pH 8.0, 1% SDS, 5 mM EDTA pH 8.0). Lysates were sonicated with Bioruptor (Diagenode) to achieve fragment sizes in the range of 200-500 bp. Lysates were diluted 1:10 in dilution buffer (50 mM Tris pH8.0, 200 mM NaCl, 5 mM EDTA pH8.0, 0.5% NP40) with protease and phosphatase inhibitors and precleared with Protein G Sepharose (GE Healthcare). Precleared lysates were incubated with 2-5 µg anti-Isl1 antibody (DSHB, clone 39.4D5) overnight at 4 °C followed by pulldown with BSA-presaturated protein G Sepharose beads. Immune complexes were washed with NaCl wash buffer (20 mM Tris pH 8.0, 500 mM NaCl, 2 mM EDTA pH 8.0, 0.1% SDS, 1% NP-40), LiCl wash buffer (20 mM Tris pH 8.0, 500 mM LiCl, 2 mM EDTA pH 8.0, 0.1% SDS, 1% NP40) and TE wash buffer (20 mM Tris pH 8.0, 2 mM EDTA pH 8.0). Protein/DNA complexes were extracted in elution buffer (20 mM Tris pH 8.0, 2 mM EDTA pH 8.0 and 2% SDS) followed by reverse crosslinking at 65 °C overnight. DNA purification was performed using phenol-chloroform and used for ChIP-qPCR analysis or ChIP- Seq.

### ChIP-qPCR

For ChIP-qPCR analysis pools of 20 cardiogenic regions of E8.25-E9 embryos or 10^6^ CPCs obtained by directed differentiation of ES cells into cardiomyocytes were used. The following antibodies were utilized: anti-Isl1 (39.4D5, DSHB), anti-Brg1 (Millipore, 07-478), anti-Gata4 (Santa Cruz, sc-1237) and anti-H3 (Abcam, ab1791) antibodies. Average threshold cycle (Ct) values were used to calculate % enrichment compared to 1% input and negative control region Ct value was used to calculate fold enrichment.

### ChIP-Seq data analysis

ChIP-Seq reads were mapped to the mouse reference genome mm9 (UCSC assembly) using the default settings of bowtie2. The MarkDuplicates.jar from Picard 1.136 was used to remove PCR artefacts. Peaks were called using MACS14^60^ with the parameter (p1e8 –nomodel –shiftsize 80) for Isl1-ChIP-Seq and Gata4-ChIP-exo (GSE72223) and (p1e3 –nomodel –shiftsize 80) for Brg1-ChIP-Seq (GSE116281). Peaks overlapping blacklist defined by ENCODE were removed. Bedtools v2.15 (intersect -wa)^61^ was used to find the common binding areas in experimental replicates and between the different *Isl1*-ChIP-Seq, Brg1-ChIP-Seq (GSE116281), Gata4-ChIP-exo (GSE72223) and ATAC-Seq datasets. Genome wide distribution of the reads was performed using ngs.plot with mm9 Ensembl (version 67) as reference annotation. ChIP-Seq peak annotation was performed using annotatePeaks.pl (default settings), which assigns each peak to the closest gene transcriptional start site (TSS). For data visualization bam files were converted to BigWig files using BamCoverage from deepTools2^62^ (-b 20 -smooth 40 -e 150 –normalizeTo1x 2150570000). Venn diagrams were generated with BioVenn online tools^63^ and protein coding genes were used for all comparisons. Taq heatmaps were created using ngsplot^64^ followed by a home made script.

### ATAC-Sequencing

Pharyngeal mesoderm and hearts were dissected from E8.75 embryos (6 somites stage). Tissues and 100.000 CPCs from two independent differentiations were resuspended in 50 uL cold lysis buffer (10 mM Tris-HCl pH 7.4, 10 mM NaCl, 3 mM MgCl_2_, 0.1% Igepal CA-630) and used for ATAC Library preparation using Tn5 Transposase from Nextera DNA Library Prep Kit (Illumina #15028211). After centrifugation (10 min, 500 g at 4 °C), cell pellet was resuspended in transposition reaction mix (25 µl TD-Buffer, 2.5 µl Tn5, 22.5 µl water) and incubated for 30 min at 37 °C with gentle mixing. Immediately following the transposition reaction, purification was carried out using MinElute PCR Purification Kit (Qiagen #28004). Amplification of Library together with indexing was performed as described in.^39^ Libraries were mixed in equimolar ratios and sequenced on NextSeq500 platform using V2 chemistry. ATAC-Seq reads were mapped with bowtie2 to mm9 mouse genome. Samtools view (-F 1804 -f2) was used to remove unmapped, mate unmapped, not primary aligned reads. The MarkDuplicates.jar from Picard 1.136 was use to remove PCR artefacts. Peaks calling was performed with MACS14^60^ (p1e5 –nomodel and –shiftsize 100), and peaks overlapping blacklist defined by ENCODE with the help of Bedtools v2.15 (intersect -wa).^61^ Similar to ChIP-Seq analysis, Bedtools v2.15 (intersect -wa) was used to obtain open chromatin regions overlapping by at least 1 bp in the replicates. BamCoverage function of deepTools2^62^ (-b 20 -smooth 40 -e 150) was used to create normalized bigwig files (reads per genome coverage, RPGC). Further, bigWigAverageOverBed (https://github.com/ENCODE-DCC/kentUtils) was used to quantify mapped reads on peaks areas. Heatmap and densitity plots were created using computeMatrix followed by plotHeatmap or plotProfile from deepTools2.^62^ To permit a comparative display of data within a deeptools profile heatmap,^62^ size factors produced by edgeR based on unified peak counts were used to scale samples to account for differences in sequencing depth, library composition, and ATAC-Seq efficiency.

ATAC-Seq reads from three wild-type, three *Isl1*^*−/−*^ *and* three *Isl1-Cre*^*−/−*^*/Brg1*^*fl/fl*^ E8.75 (6 somites) embryos and ESC-derived CPCs from two independent differentiations were used for further analysis.

### Additional bioinformatic analysis

Gene ontology analysis was performed using DAVID Bioinformatics Resources 6.8. The *Isl1* regulated gene network presented in Fig. 3d was built by submitting a curated list of *Isl1* primary downstream targets to the GeneMANIA web based software. The network was subdivided into transcription factors, signaling molecules, muscle structural and contractile genes involved in heart development and visualized using Cytoscape.

Intersection of RNA-Seq and ChIP-Seq data was done by gene name. Intersection of ATAC-Seq and ChIP-Seq peaks was performed with Bedtools v2.15 (intersect -wa) to identify open chromatin regions overlapping at least 1 bp with *Isl1*, Brg1 or GATA4 bound sequences.

Gene list in GO terms related to cardiac muscle cell differentiation, endothelial cell differentiation and smooth muscle cell development/ differentiation was obtained from (http://amigo.geneontology.org/amigo/landing). DeepTools2^62^ was used to plot ATAC-Seq reads covering TSS of genes within the above mentioned gene lists.

### Protein expression and purification

6XHis tagged *Isl1* DNA binding homeodomain (HD) protein^65^ was expressed in E. Coli Rosetta DE3 cells and purified using Ni-NTA agarose (Qiagen, 30210) under native conditions. Purified *Isl1* protein was further dialyzed in TE (10 mM Tris pH 8.0 and 1 mM EDTA pH 8.0) overnight using Spectra/Por 3 basic dialysis unit (Cat#132720), aliquoted and stored at −80 °C.

Nuclear extracts from Nkx2.5 overexpressing HEK293T cells were prepared as follows: cells were scraped in 400 ml of buffer A (10 mM HEPES pH7.9, 1.5 mM MgCl_2_, 10 mM KCl, 0.5 mM DTT, protease inhibitor cocktail, and 0.5 mM PMSF) and incubated on ice for 15 min. After centrifugation, the cell pellet was resuspended in 150 ml of buffer B (20 mM HEPES pH7.9, 1.5 mM MgCl_2_, 420 mM NaCl, 0.2 mM EDTA, 25% v/v Glycerol, protease inhibitor cocktail, and 0.5 mM PMSF) and incubated on ice with vigorous agitation for 40 min. Nuclear extracts were recovered by centrifugation for 15 min at 13,000 rpm and protein concentration was determined by BCA method (Thermo Fisher Scientific Cat# 23225). Nuclear extracts were aliquoted and stored at −80 °C.

### Nucleosome assembly

162 bp *Ttn* promoter DNA was PCR amplified from mouse genomic DNA and cloned into pJET vector (Thermo Fisher Scientific Cat# K1231). The genomic DNA used corresponds to the following sequence:

CGGTGTCTGCTATCACTCAGTTTCATCCCTTTCTTCCCAAATGGCCAGTTTCATGTTGTGTATCTTGACAGTGACTCCCAACCAGAGACTCCTGTTAAGGGAACTTGTCAGTCATAATAGACAATAAGTGAACTTCTTGTTCGGGGTGCTCAATAATTTGCT

Using biotin labeled PCR primers *Ttn* DNA was amplified and purified using PCR cleanup kit (Qiagen, 28004). Purified human recombinant histone H2A/H2B dimers (NEB#M2508S) and H3/H4 tetramers (NEB, M2509S) were purchased from NEB. DNA was assembled into nucleosomes as described in.^1^ Briefly, 2 µg of biotin labeled *Ttn* DNA was mixed with equimolar ratios of H2A/H2B dimer and H3/H4 tetramers at 1:1.4 DNA:Histone Octamer molar ratio in buffer containing 10 mM Tris-HCl pH 8.0, 5 M urea, 2 M NaCl, 1 mM EDTA and 0.1 mg/ml BSA. Nucleosome assembly was carried out using the salt gradient dialysis method with a buffer series containing 2 M, 1.5 M, 1 M, 0.8 M and 0.6 M, NaCl in 10 mM Tris pH 8.0, 1 mM EDTA pH 8.0, 5 M Urea and 10 mM β-mercaptoethanol at 4 °C. Buffer exchange was performed every 4 h. Finally, the nucleosomes were dialyzed in 0.6 M NaCl or 0.1 M NaCl, 10 mM Tris pH 8.0, 1 mM EDTA pH 8.0 and 1 mM β-mercaptoethanol buffer without urea for 4 h and O/N respectively at 4 °C.

### Binding reactions and Electrophoretic mobility shift assays

Biotin labeled free *Ttn* DNA, nucleosomal *Ttn* DNA and increasing concentrations of *Isl1* protein (0 to 1 µM) or nuclear extract from Nkx2.5 overexpressing cells were incubated in binding buffer containing 40 mM KCl, 15 mM HEPES pH8.0, 1 mM EDTA pH8.0, 0.5 mM DTT, 2 mM MgCl_2_, 5% glycerol, 10 ng/µl poly dI/dC and 0.1 mg/ml BSA for 30 min at RT. The free and bound DNA was resolved on 4% PAGE gel in 0.5X Tris borate-EDTA (TBE) and transferred onto a Biodyne® B Nylon Membrane (Thermo Fisher Scientific Cat# 77016) in 0.5X TBE for 1 h at 350 mAmp. Detection of the biotin-labeled DNA was performed with Chemiluminescent Nucleic Acid Detection Module (Thermo Fisher Scientific Cat# 89880) according to manufacturer’s instructions. For competition assays 0.5 µM *Isl1* protein was used in combination with 80-fold molar excess of specific (Insulin enhancer; 5′CCCTTGTTAAGACTCTAATTACCCTAG3′) and nonspecific (NS; 5′CAAGCAAAACAAACCA3′) double stranded oligos as competitors. Representative results of three independent nucleosomal assembly, binding and competition assays are presented.

### Statistics and reproducibility

All experiments were performed at least three independent times (unless noted otherwise) and respective data were used for statistical analyses. Differences between groups were assessed using a two-tailed Student’s t-test in Microsoft Excel. *p* values represent significance *p* ≤ 0.05*, *p* ≤ 0.01**, *p* ≤ 0.005***.

## Supplementary information

Supplementary information, Figure S1

Supplementary information, Figure S2

Supplementary information, Figure S3

Supplementary information, Figure S4

Supplementary information, Figure S5

Supplementary information, Figure S6

Supplementary information, Figure S7

Supplementary information, Figure S8

Supplementary information, Table S1

Supplementary information, Table S2

Supplementary information, Table S3

Supplementary information, Table S4

Supplementary information, Table S5

Supplementary information, Table S6

Supplementary information, Table S7

Supplementary information, Table S8

## Data Availability

Sequencing data (ChIP-Seq, RNA-Seq, ATAC-Seq) have been deposited in the Gene Expression Omnibus (GEO) under accession code GSE80383. All other data supporting the findings of this study are available from the corresponding authors on request.
