# Supplementary material for: Pioneering function of Isl1 in the epigenetic control of cardiomyocyte cell fate
Source: Cell Res. 2019 Apr 25;29(6):486–501. doi: 10.1038/s41422-019-0168-1 (PMC6796926; doi:10.1038/s41422-019-0168-1)
Supplement: Supplementary file 1 — Supplementary information, Figure S1 [file 41422_2019_168_MOESM1_ESM.pdf]

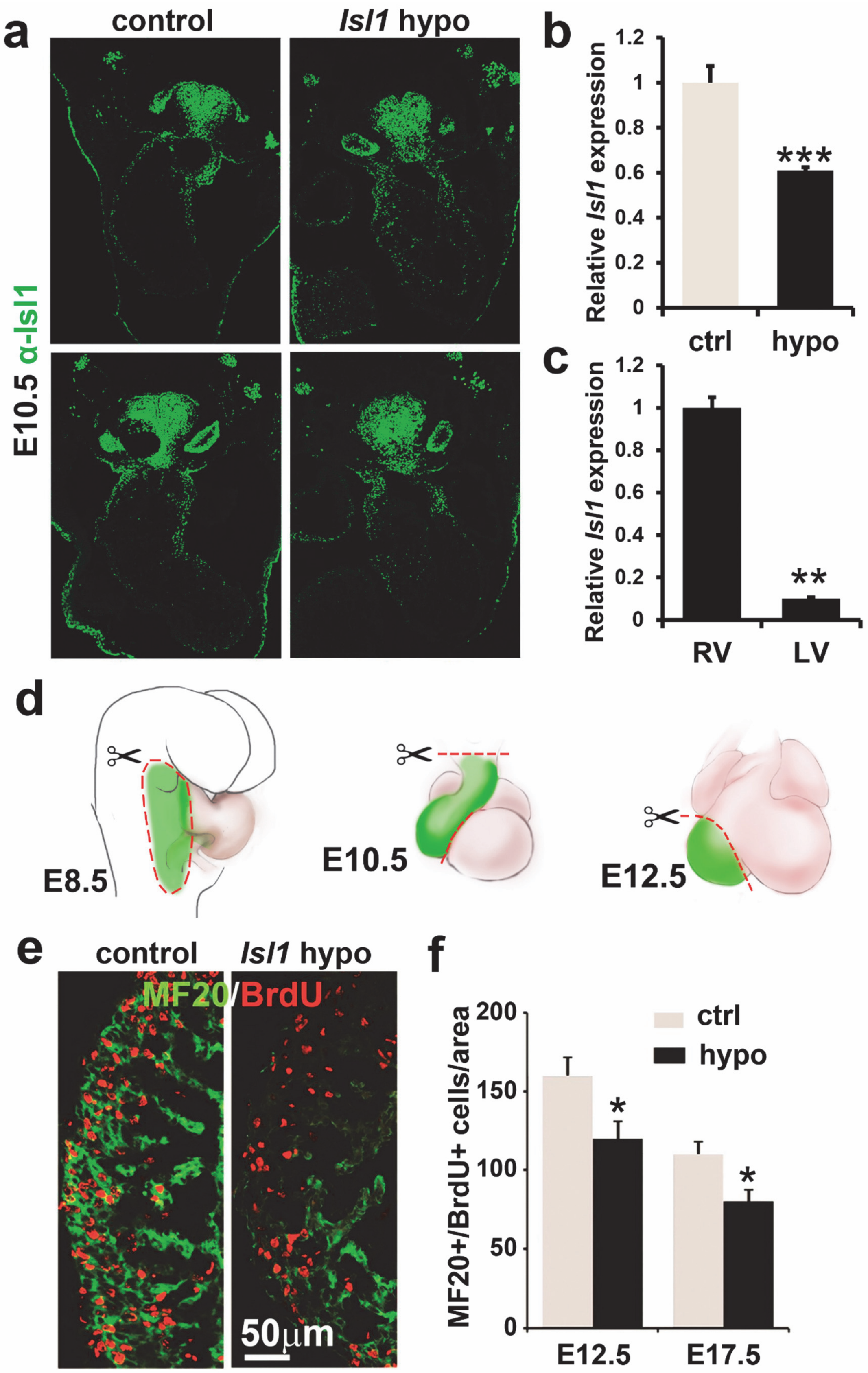
a
control
Isl1 hypo
E10.5 α-Isl1
b
Relative Isl1 expression
1.2
1
0.8
0.6
0.4
0.2
0
***
ctrl
hypo
c
Relative Isl1 expression
1.2
1
0.8
0.6
0.4
0.2
0
**
RV
LV
d
E8.5
E10.5
E12.5
e
control
Isl1 hypo
MF20/BrdU
50μm
f
MF20+/BrdU+ cells/area
200
150
100
50
0
ctrl
hypo
*
*
E12.5
E17.5

Supplementary information, Figure S1 | Reduced cardiomyocyte proliferation and MF20 immunoreactivity in *Isl1* hypomorphic embryos. (a) Immunostaining of control and *Isl1* hypomorphic embryos with anti Isl1 antibody. **(b)** Relative *Isl1* mRNA expression in OFT+RV of control and *Isl1* hypomorphic embryos. **(c)** Relative *Isl1* mRNA expression in RV versus LV of E10.5 wild-type embryos. **(d)** Schematic representation of the dissection procedures used in the study: E8.5, pharyngeal mesoderm labeled in green; E10.5, OFT+RV in green; E12.5, RV in green. **(e)** Co-immunostaining of control and *Isl1* hypomorphic right ventricles (hypo) with BrdU and MF20 at E12.5 showing a marked reduction in the number of BrdU+ cardiomyocytes (MF20+) and MF20 immunoreactivity. **(f)** Quantification of BrdU+/ MF20+ cells in RV free wall at E12.5 and E17.5. Data represent mean ± SEM, n=4.
