# Supplementary material for: Pioneering function of Isl1 in the epigenetic control of cardiomyocyte cell fate
Source: Cell Res. 2019 Apr 25;29(6):486–501. doi: 10.1038/s41422-019-0168-1 (PMC6796926; doi:10.1038/s41422-019-0168-1)
Supplement: Supplementary file 2 — Supplementary information, Figure S2 [file 41422_2019_168_MOESM2_ESM.pdf]

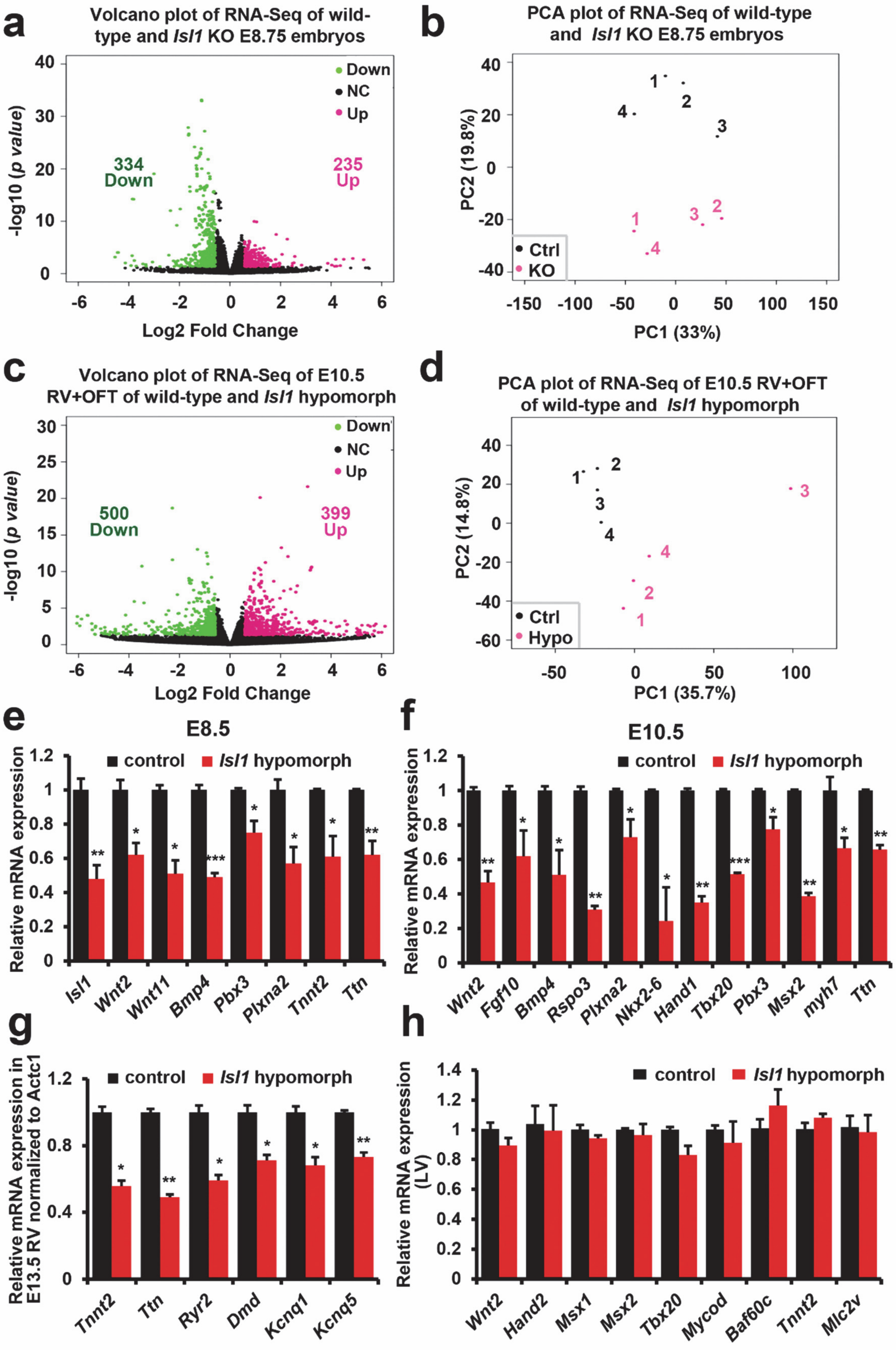

a
Volcano plot of RNA-Seq of wild-type and Isl1 KO E8.75 embryos
334 Down
235 Up
Down
NC
Up
-log10 (p value)
Log2 Fold Change
b
PCA plot of RNA-Seq of wild-type and Isl1 KO E8.75 embryos
PC2 (19.8%)
PC1 (33%)
Ctrl
KO
c
Volcano plot of RNA-Seq of E10.5 RV+OFT of wild-type and Isl1 hypomorph
500 Down
399 Up
d
PCA plot of RNA-Seq of E10.5 RV+OFT of wild-type and Isl1 hypomorph
PC2 (14.8%)
PC1 (35.7%)
Ctrl
Hypo
e
E8.5
control
Isl1 hypomorph
Relative mRNA expression
Isl1
Wnt2
Wnt11
Bmp4
Pbx3
Plxna2
Tnnt2
Ttn
f
E10.5
Wnt2
Fgf10
Bmp4
Rspo3
Plxna2
Nkx2-6
Hand1
Tbx20
Pbx3
Msx2
myh7
Ttn
g
Relative mRNA expression in E13.5 RV normalized to Actc1
Tnnt2
Ttn
Ryr2
Dmd
Kcnq1
Kcnq5
h
Relative mRNA expression (LV)
Wnt2
Hand2
Msx1
Msx2
Tbx20
Mycod
Baf60c
Tnnt2
Mlc2v

Supplementary information, Figure S2 | Gene expression changes in control and *Isl1* hypomorphic or *Isl1* knockout embryos. (a) Volcano plot of RNA-Seq analysis of dissected pharyngeal mesoderm and hearts of wild-type and E8.75 Isl1 knockout embryos. Down- and upregulated genes (n=4, fold change> 1.5; log2 fold change < -0.58, >0.58; p-value < 0.05) are reported as green and magenta dots, respectively. Not differentially expressed genes are represented as black dots. **(b)** Principal component analysis (PCA) of variance stabilized transformed RNA-Seq read counts of top 10,000 genes of wild-type and E8.75 *Isl1* knockout embryos (n=4). **(c)** Volcano plot of RNA-Seq analysis of dissected OFT and RV of E10.5 wild-type and *Isl1* hypomorphic embryos. Down- and upregulated genes (n=4, fold change> 1.5; log2 fold change < -0.58, >0.58; p-value < 0.05) are reported as green and magenta dots, respectively. Not differentially expressed genes are represented as black dots. **(d)** Principal component analysis (PCA) of variance stabilized transformed RNA-Seq read counts of top 10,000 genes of OFT and RV of E10.5 wild-type and *Isl1* hypomorphic embryos (n=4). **(e, f)** Relative mRNA expression of selected Isl1 bound genes involved in OFT septation and cardiomyocyte (CM) contraction and structure in dissected pharyngeal and outflow tract regions of E8.5 **(e)** or OFT and RV of E10.5 *Isl1* hypomorphic embryos **(f)**. Data are mean+SEM, n=4. **(g)** Relative mRNA expression of selected Isl1 bound cardiomyocyte (CM) contraction and structural genes in dissected RV of E13.5 *Isl1* hypomorphic embryos. To account for the reduced RV myocardium wall thickness further normalization to cardiac *Actc1*, which is not Isl1-primary target, was performed. **(h)** Relative mRNA expression of selected Isl1 bound genes in dissected LV of E12.5 control and *Isl1* hypomorphic embryos. Data represent mean ± SEM, n=3.
