# Supplementary material for: Pioneering function of Isl1 in the epigenetic control of cardiomyocyte cell fate
Source: Cell Res. 2019 Apr 25;29(6):486–501. doi: 10.1038/s41422-019-0168-1 (PMC6796926; doi:10.1038/s41422-019-0168-1)
Supplement: Supplementary file 3 — Supplementary information, Figure S3 [file 41422_2019_168_MOESM3_ESM.pdf]

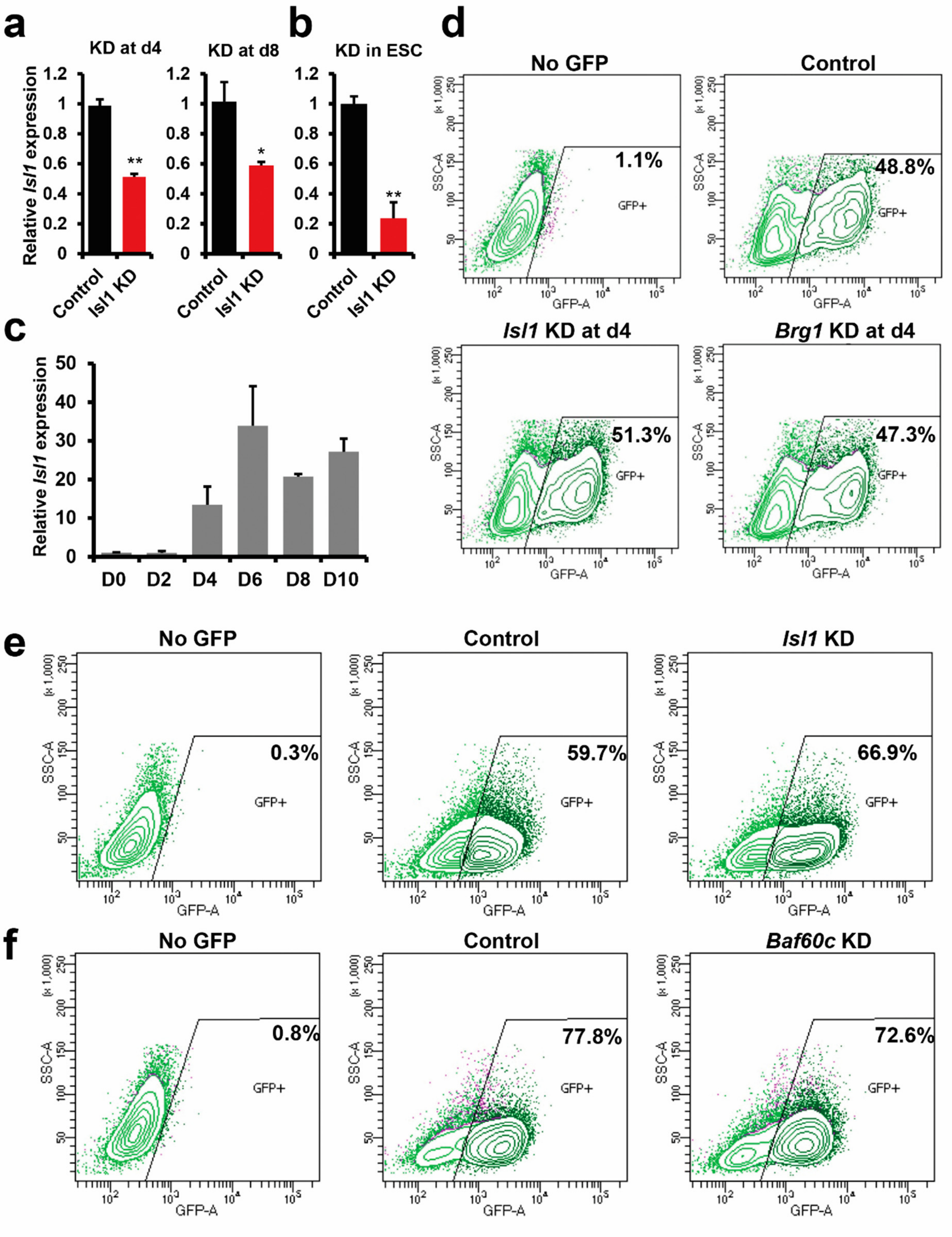

a
KD at d4
KD at d8
Relative Isl1 expression
1.2
1
0.8
0.6
0.4
0.2
0
**
*
Control
Isl1 KD
b
KD in ESC
**
c
Relative Isl1 expression
50
40
30
20
10
0
D0
D2
D4
D6
D8
D10
d
No GFP
Control
Isl1 KD at d4
Brg1 KD at d4
1.1%
48.8%
51.3%
47.3%
GFP+
SSC-A
GFP-A
e
No GFP
Control
Isl1 KD
0.3%
59.7%
66.9%
f
No GFP
Control
Baf60c KD
0.8%
77.8%
72.6%

Supplementary information, Figure S3 | Isl1 and Baf60c knockdown efficiency and differentiation efficiency of ESCs after *Isl1*, *Baf60c* or *Brg1* KD. **(a)** Relative *Isl1* expression in cardiomyocytes after knockdown of Isl1 at distinct stages of directed cardiac differentiation. **(b)** Relative Isl1 expression in CPCs derived from ESC expressing control or *Isl1* shRNA. **(c)** Relative *Isl1* expression in the course of directed cardiac differentiation. **(d)** Percentage of Nkx2-5–GFP+ cells at CPC stage after *Isl1* or *Brg1* knockdown at d4. **(e)** Percentage of Nkx2-5–GFP+ cells, differentiated from E14, control Nkx2-5–GFP ESCs or Nkx2-5–GFP ESCs expressing shRNA against *Isl1*, at CPC stage. **(f)** Percentage of Nkx2-5–GFP+ cells, differentiated from E14, control Nkx2-5–GFP ESCs or Nkx2-5–GFP ESCs expressing shRNA against *Baf60c*, at CPC stage.
