# Supplementary material for: Pioneering function of Isl1 in the epigenetic control of cardiomyocyte cell fate
Source: Cell Res. 2019 Apr 25;29(6):486–501. doi: 10.1038/s41422-019-0168-1 (PMC6796926; doi:10.1038/s41422-019-0168-1)
Supplement: Supplementary file 4 — Supplementary information, Figure S4 [file 41422_2019_168_MOESM4_ESM.pdf]

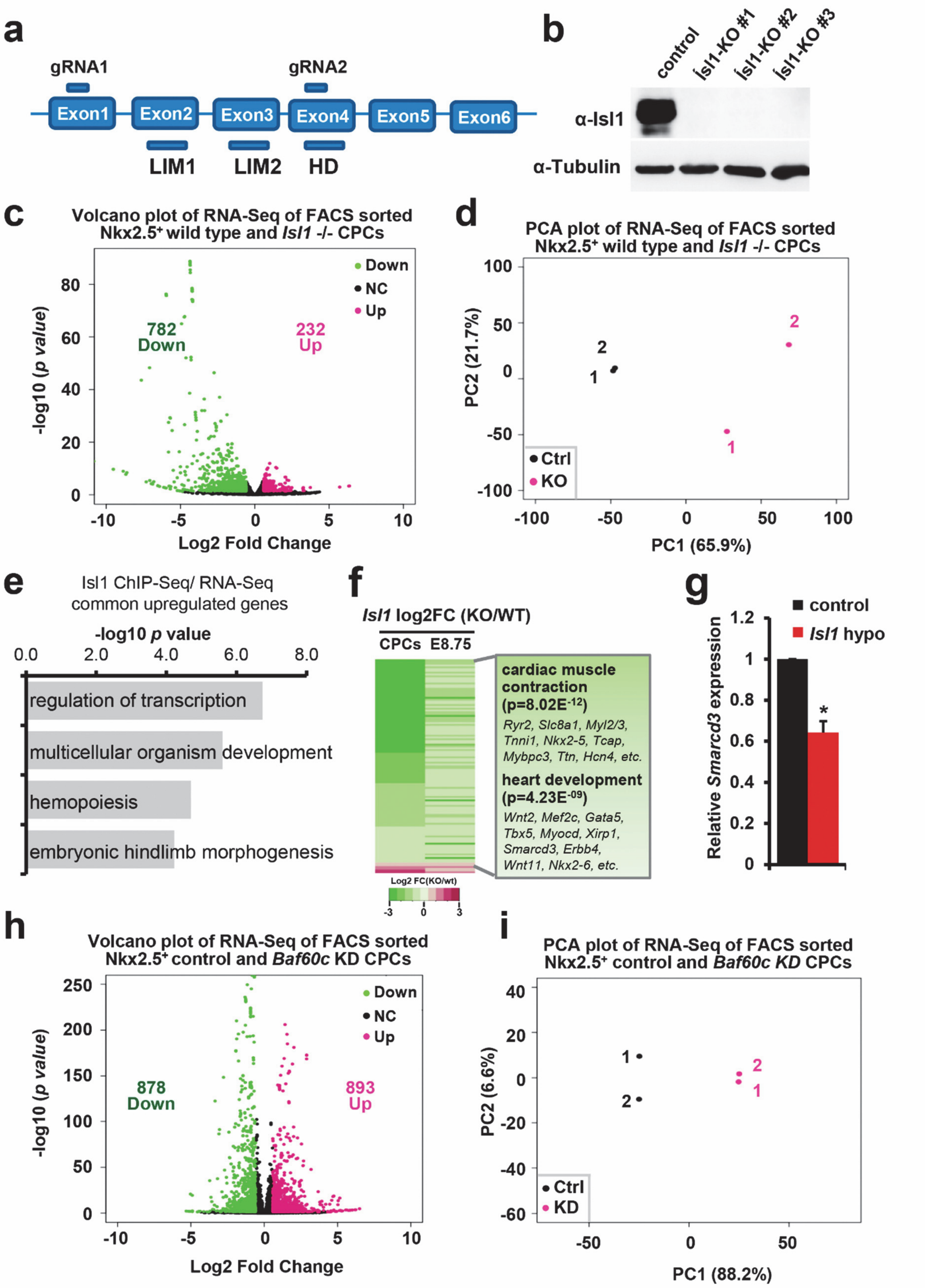
a
gRNA1
gRNA2
Exon1
Exon2
Exon3
Exon4
Exon5
Exon6
LIM1
LIM2
HD
b
control
Isl1-KO #1
Isl1-KO #2
Isl1-KO #3
α-Isl1
α-Tubulin
c
Volcano plot of RNA-Seq of FACS sorted
Nkx2.5+ wild type and Isl1 -/- CPCs
Down
NC
Up
782
Down
232
Up
-log10 (p value)
Log2 Fold Change
d
PCA plot of RNA-Seq of FACS sorted
Nkx2.5+ wild type and Isl1 -/- CPCs
Ctrl
KO
PC2 (21.7%)
PC1 (65.9%)
e
Isl1 ChIP-Seq/ RNA-Seq
common upregulated genes
-log10 p value
regulation of transcription
multicellular organism development
hemopoiesis
embryonic hindlimb morphogenesis
f
Isl1 log2FC (KO/WT)
CPCs E8.75
cardiac muscle contraction
(p=8.02E-12)
Ryr2, Slc8a1, Myl2/3, Tnni1, Nkx2-5, Tcap, Mybpc3, Ttn, Hcn4, etc.
heart development
(p=4.23E-09)
Wnt2, Mef2c, Gata5, Tbx5, Myocd, Xirp1, Smarcd3, Erbb4, Wnt11, Nkx2-6, etc.
Log2 FC(KO/wt)
g
control
Isl1 hypo
Relative Smarcd3 expression
h
Volcano plot of RNA-Seq of FACS sorted
Nkx2.5+ control and Baf60c KD CPCs
Down
NC
Up
878
Down
893
Up
-log10 (p value)
Log2 Fold Change
i
PCA plot of RNA-Seq of FACS sorted
Nkx2.5+ control and Baf60c KD CPCs
Ctrl
KD
PC2 (6.6%)
PC1 (88.2%)

Supplementary information, Figure S4 | Gene expression changes in control, *Isl1*-/- and Baf60c KD mESC-derived CPCs. **(a)** Schematic representation of the strategy used to generate *Isl1*-/- mESCs using the CRISPR/Cas9 gene editing technology. **(b)** Western blot analysis of protein extracts from control and *Isl1*-/- CPCs (day 5) derived from control and *Isl1*-/- mESCs. Tubulin served as a loading control. **(c)** Volcano plot of RNA-Seq analysis of FACS-sorted Nkx2.5^{+} CPCs derived from two independent control and *Isl1*-/- mESC clones. Down- and upregulated genes (n=2, fold change> 1.5; log2 fold change < -0.58, >0.58; p-value < 0.05) are reported as green and magenta dots, respectively. Not differentially expressed genes are represented as black dots. **(d)** Principal component analysis (PCA) of variance stabilized transformed RNA-Seq read counts of top 10,000 genes of control and *Isl1*-/- Nkx2.5^{+} CPCs (n=2). **(e)** GO terms enriched in genes bound by Isl1 in ESC-derived CPCs and upregulated in *Isl1*-/- CPCs. **(f)** Heatmap of genes bound by Isl1 and deregulated in both RNA-Seq analysis, of dissected pharyngeal mesoderm and hearts of wild-type and E8.75 Isl1 knockout embryos (n=4) and of control and *Isl1*-/- Nkx2.5^{+} CPCs (n=2) (fold change > 1.5, p < 0.05). Representative genes and enriched GO terms are presented on the right side**.** **(g)** Relative mRNA expression of Baf60c (*Smarcd3*) in dissected OFT and RV of E10.5 control and Isl1 hypomorphic embryos. Data represent mean ± SEM, n=3. **(h)** Volcano plot of RNA-Seq analysis of FACS-sorted control and *Baf60c* KD Nkx2.5^{+} CPCs. Down- and upregulated genes (n=2, fold change> 1.5; log2 fold change < -0.58, >0.58; p-value < 0.05) are reported as green and magenta dots, respectively. Not differentially expressed genes are represented as black dots. **(i)** Principal component analysis (PCA) of variance stabilized transformed RNA-Seq read counts of top 10,000 genes of control and *Baf60c* KD Nkx2.5^{+} CPCs (n=2).
