# Supplementary material for: Pioneering function of Isl1 in the epigenetic control of cardiomyocyte cell fate
Source: Cell Res. 2019 Apr 25;29(6):486–501. doi: 10.1038/s41422-019-0168-1 (PMC6796926; doi:10.1038/s41422-019-0168-1)
Supplement: Supplementary file 5 — Supplementary information, Figure S5 [file 41422_2019_168_MOESM5_ESM.pdf]

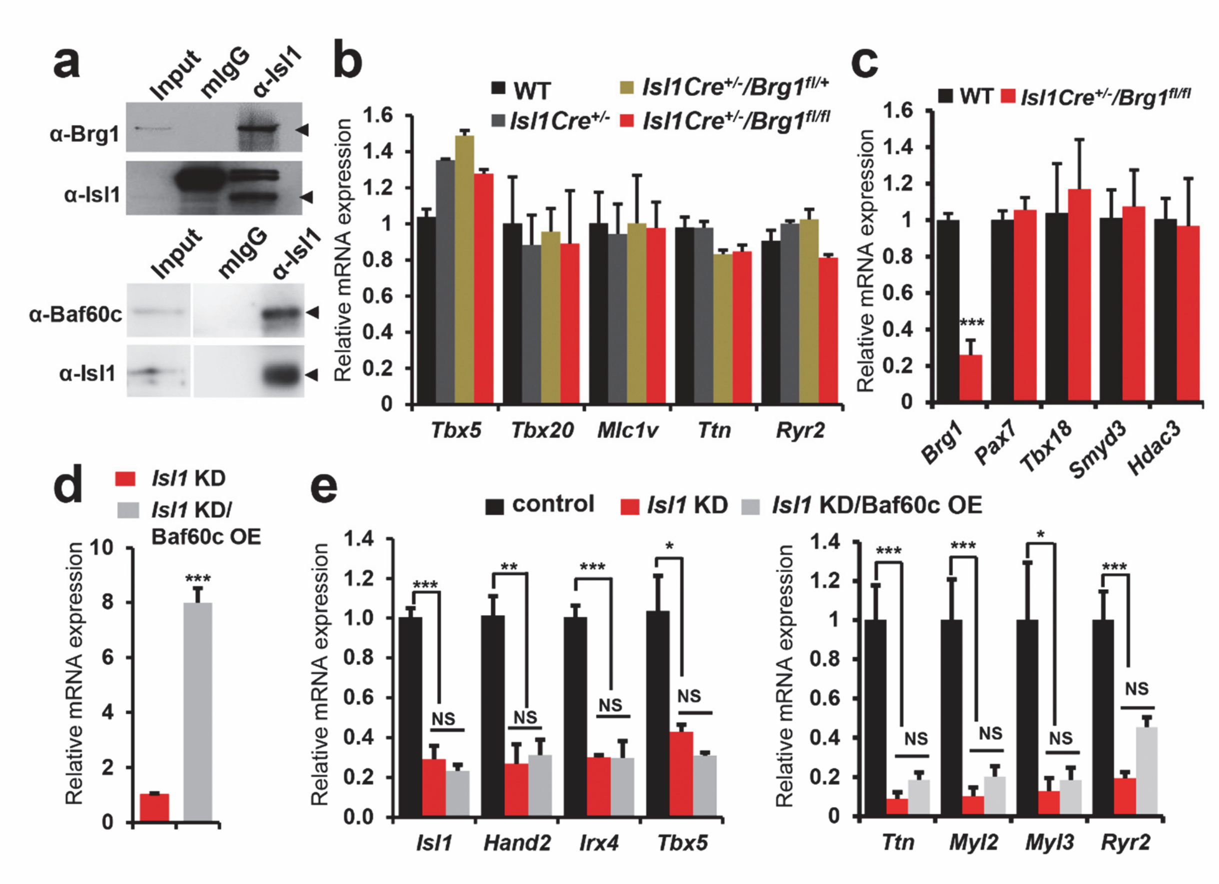

Supplementary information, Figure S5 | Isl1 works in concert with the Brg1-based SWI/SNF complex to regulate its target gene expression. (a) Co-immunoprecipitation showing interaction between Isl1 and Brg1 or Baf60c in mESC-derived CPCs. **(b)** Relative mRNA expression levels of Isl1/Brg1-Baf60c common targets in dissected left ventricle of E10.5 wild-type, *Isl1Cre*$^{+/-}$, *Isl1Cre*$^{+/-}$*/Brg1*$^{+/fl}$ and *Isl1Cre*$^{+/-}$*/Brg1*$^{fl/fl}$ embryos. Data are mean+SEM, n=3. **(c)** Relative mRNA expression levels of *Brg1* and genes not regulated by Isl1 in dissected OFT and RV of wild-type, *Isl1-Cre*$^{+/-}$, *Isl1-Cre*$^{+/-}$*/Brg1*$^{+/fl}$ and *Isl1-Cre*$^{+/-}$*/Brg1*$^{fl/fl}$ E10.5 embryos. Data are mean ± SEM, n=3. **(d)** Relative *Baf60c* expression in *Isl1* KD and Isl1KD/Baf60c overexpressing (Isl1KD/Baf60c OE) CPCs. **(e)** Relative mRNA expression levels of Isl1/Brg1-Baf60c common targets in control, *Isl1* KD and Isl1KD/Baf60c overexpressing (Isl1KD/Baf60c OE) d5 EBs (left panel) or d9 EBs (right panel).
