# Supplementary material for: Pioneering function of Isl1 in the epigenetic control of cardiomyocyte cell fate
Source: Cell Res. 2019 Apr 25;29(6):486–501. doi: 10.1038/s41422-019-0168-1 (PMC6796926; doi:10.1038/s41422-019-0168-1)
Supplement: Supplementary file 6 — Supplementary information, Figure S6 [file 41422_2019_168_MOESM6_ESM.pdf]

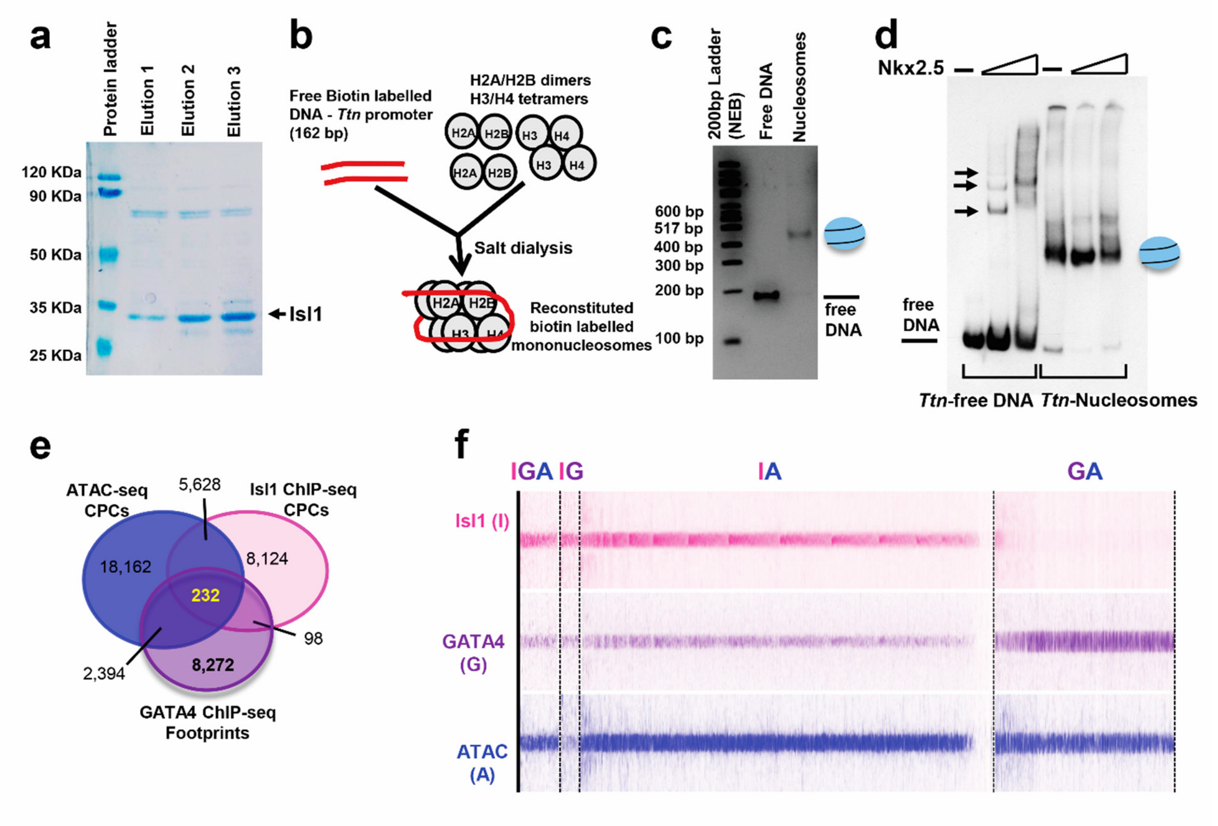

Supplementary information, Figure S6 | Isl1 recognizes its DNA binding motif on DNA wrapped around nucleosomes and Isl1 binding correlates with sites of open chromatin. (a-c) Isl1 purification and nucleosome assembly. SDS PAGE gel stained with Coomassie brilliant blue showing purified recombinant Isl1-HD protein **(a)**. Scheme illustrating the assembly of nucleosomes on 162bp *Ttn* genomic DNA fragment **(b)**. Free and nucleosomal *Ttn* DNA visualized by ethidium bromide staining **(c)**. **(d)** EMSA with increasing amount of nuclear extract from Nkx2-5 overexpressing HEK293T cells and free *Ttn* DNA fragment (left, lane 1-3) and the same fragment assembled into nucleosomes (right, lane 4-6). **(e)** Overlap of all Isl1 ChIP-Seq, GATA4 ChIP-exo average footprints[32] and ATAC-Seq peaks (top). **(f)** Heatmap of mapped reads of Isl1 ChIP-Seq, GATA4 ChIP-exo average footprints[32] and ATAC-Seq at ± 2 kb around peak midpoints of Isl1, GATA4 and ATAC (IGA); Isl1 and GATA4 (IG); Isl1 and ATAC (IA); GATA4 and ATAC (GA) occupancy groups.
