# Supplementary material for: Pioneering function of Isl1 in the epigenetic control of cardiomyocyte cell fate
Source: Cell Res. 2019 Apr 25;29(6):486–501. doi: 10.1038/s41422-019-0168-1 (PMC6796926; doi:10.1038/s41422-019-0168-1)
Supplement: Supplementary file 7 — Supplementary information, Figure S7 [file 41422_2019_168_MOESM7_ESM.pdf]

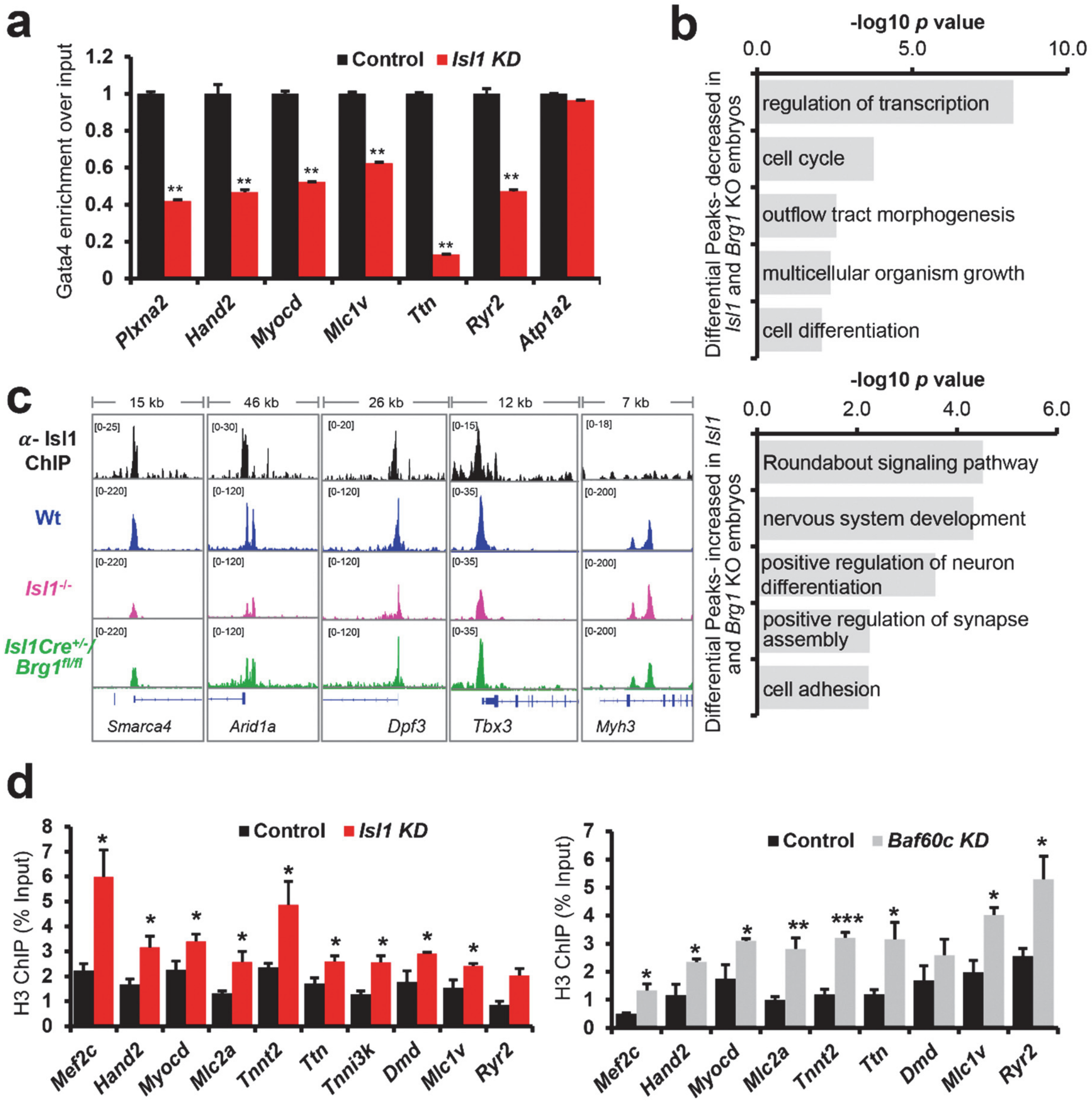

Supplementary information, Figure S7 | Isl1 and the Brg1-Baf60c complex induce chromatin reorganization in CPCs. (a) ChIP-qPCR to analyze GATA4 occupancy at Isl1- bound sequences in control and Isl1 KD CPCs. *Atp1a2*, a gene not bound by Isl1, serves as a negative control. **(b)** GO terms enriched in ATAC-Seq peaks decreased (top panel) or increased (lower panel) in both *Isl1*$^{-/-}$ and *Isl1-Cre*$^{+/-}$*Brg1*$^{fl/fl}$ embryos versus control embryos (n=3). **(c)** Examples of genes showing decreased ATAC-Seq signals at Isl1 binding sites in both *Isl1*$^{-/-}$ and *Isl1-Cre*$^{+/-}$ *Brg1*$^{fl/fl}$ embryos (*Smarca4*, *Arid1a*), genes bound by Isl1 with decreased chromatin accessibility

only in *Isl1*$^{-/-}$ knockout embryos (*Tbx3, Dpf3*) and genes not bound by Isl1 showing no change in ATAC signal in both *Isl1*$^{-/-}$ and *Isl1-Cre*$^{+/-}$*Brg1*$^{fl/fl}$ embryos (*Myh3*). Genome tracks of Isl1 ChIP-Seq and ATAC-Seq signals of wild-type, *Isl1*$^{-/-}$ and *Isl1-Cre*$^{+/-}$*Brg1*$^{fl/fl}$ embryos are presented. **(d)** ChIP-qPCR analysis of relative H3 occupancy at selected Isl1 target genes in control and *Isl1* knockdown CPCs (left) and control and *Baf60c* knockdown CPCs (right). Data are mean ± SEM, n=3.
