# Supplementary material for: Pioneering function of Isl1 in the epigenetic control of cardiomyocyte cell fate
Source: Cell Res. 2019 Apr 25;29(6):486–501. doi: 10.1038/s41422-019-0168-1 (PMC6796926; doi:10.1038/s41422-019-0168-1)
Supplement: Supplementary file 8 — Supplementary information, Figure S8 [file 41422_2019_168_MOESM8_ESM.pdf]

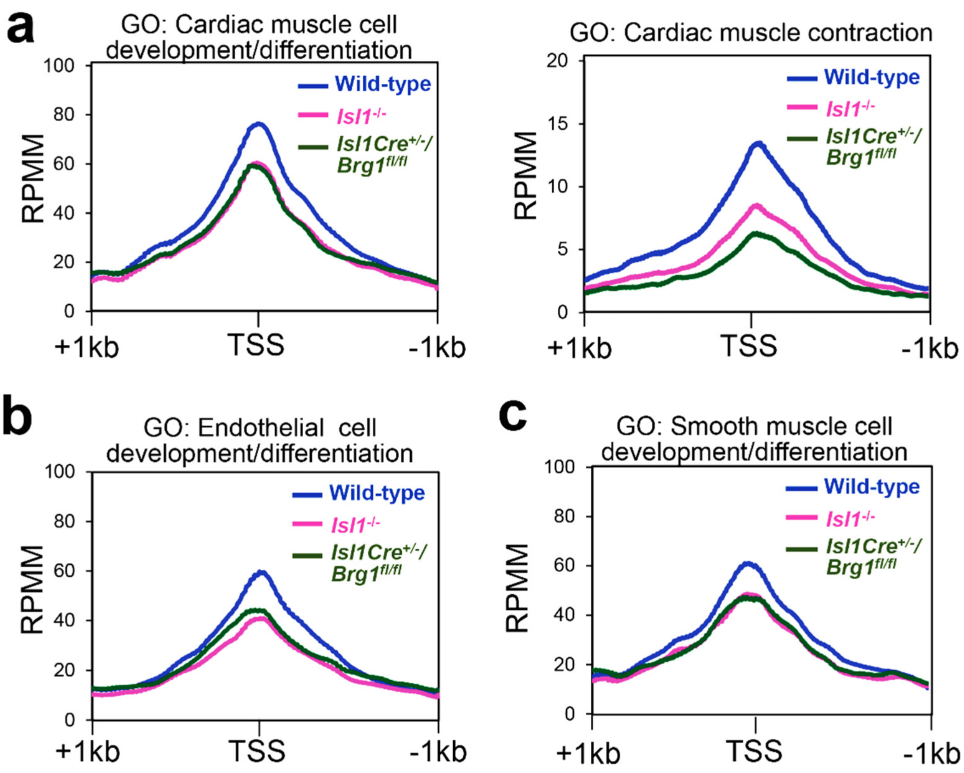

Supplementary information, Figure S8 | Isl1 and the Brg1-Baf60c complex induce chromatin reorganization in CPCs. (a-d) Average ATAC-Seq tag intensities at TSS+/- 1kb of cardiac development and differentiation genes **(a**, left panel**)**, cardiac contraction genes **(a**, right panel**)**, as well as smooth muscle **(b)** and endothelial **(c)** development and differentiation genes in E8.75 wild-type, *Isl1*$^{-/-}$ and *Isl1-Cre*$^{+/-}$*Brg1*$^{fl/fl}$ embryos (n=3).
